# Novel Multifunctional Ascorbic Triazole Derivatives for Amyloidogenic Pathway Inhibition, Anti-Inflammation, and Neuroprotection

**DOI:** 10.3390/molecules26061562

**Published:** 2021-03-12

**Authors:** Jutamas Jiaranaikulwanitch, Hataichanok Pandith, Sarin Tadtong, Phanit Thammarat, Supat Jiranusornkul, Nattapong Chauthong, Supitcha Nilkosol, Opa Vajragupta

**Affiliations:** 1 Innovation Center for Holistic Health, Nutraceuticals, and Cosmeceuticals, Faculty of Pharmacy, Chiang Mai University, Chiang Mai 50200, Thailand; 2 Department of Pharmaceutical Sciences, Faculty of Pharmacy, Chiang Mai University, Chiang Mai 50200, Thailand; phanit.thamma@cmu.ac.th (P.T.); supat.jira@cmu.ac.th (S.J.); nattapong.chau@gmail.com (N.C.); supitchanilkosol@gmail.com (S.N.); 3 Department of Biology, Faculty of Science, Chiang Mai University, Chiang Mai 50200, Thailand; hataichanok.p@cmu.ac.th; 4 Department of Pharmacognosy, Faculty of Pharmacy, Srinakharinwirot University, Nakhonnayok 26120, Thailand; sarin@g.swu.ac.th; 5 Office of Research Affairs, Faculty of Pharmaceutical Sciences, Chulalongkorn University, Bangkok 10330, Thailand; opa.v@chula.ac.th

**Keywords:** ascorbic derivatives, BACE1 inhibitor, amyloid aggregation inhibition, antioxidant, neuroprotective, anti-inflammation

## Abstract

Alzheimer’s disease (AD) is a common neurodegenerative disorder. The number of patients with AD is projected to reach 152 million by 2050. Donepezil, rivastigmine, galantamine, and memantine are the only four drugs currently approved by the United States Food and Drug Administration for AD treatment. However, these drugs can only alleviate AD symptoms. Thus, this research focuses on the discovery of novel lead compounds that possess multitarget regulation of AD etiopathology relating to amyloid cascade. The ascorbic acid structure has been designated as a core functional domain due to several characteristics, including antioxidant activities, amyloid aggregation inhibition, and the ability to be transported to the brain and neurons. Multifunctional ascorbic derivatives were synthesized by copper (I)-catalyzed azide–alkyne cycloaddition reaction (click chemistry). The in vitro and cell-based assays showed that compounds **2c** and **5c** exhibited prominent multifunctional activities as beta-secretase 1 inhibitors, amyloid aggregation inhibitors, and antioxidant, neuroprotectant, and anti-inflammatory agents. Significant changes in activities promoting neuroprotection and anti-inflammation were observed at a considerably low concentration at a nanomolar level. Moreover, an in silico study showed that compounds **2c** and **5c** were capable of being permeated across the blood–brain barrier by sodium-dependent vitamin C transporter-2.

## 1. Introduction

Alzheimer’s disease (AD) is the main cause of dementia. It is a common neurodegenerative disorder, with significant attributes relating to memory loss and cognitive function impairments. It has negative impacts on patients’ thinking abilities, behaviors, and daily routine activities. In 2020, over 50 million people have dementia, and the number of patients will reach 82 million in 2030 and 152 million in 2050 [1]. Amyloid cascade is one of several biological pathways that significantly leads to the onset and progression of AD. Considerable evidence has indicated that each form of amyloid-β peptide aggregation contributes to AD etiopathology. This includes the induction of toxic conditions to neurons such as lipid peroxidation [2], depolarization of synaptic membranes [2], production of free radicals [3], induction of inflammatory response, and mediation of proinflammatory cytokines [4]. Moreover, amyloid-β peptides can damage synapse-, neurite-, and neuron-producing serotonin, epinephrine, glutamate, and acetylcholine neurotransmitters [5].

Beta-secretase 1 (BACE1) and gramma-secretase are important enzymes that cleave the amyloid-β precursor protein (APP), leading to the generation and accumulation of amyloid-β peptides in neurons. Aggregations of these peptides set off the oligomerization processes that produce amyloid-β peptide oligomers. The oligomers then react with the reactive metals in the brain; as a result, they produce neurotoxic reactive oxygen species (ROS). Moreover, amyloid-β peptide oligomers have been reported to induce neuronal inflammation in the AD brain via the upregulation of inflammation-related genes, cyclooxygenase-2 (*COX-2*) and inducible nitric oxide synthase (*iNOS*) [6,7]. Altogether, increasing ROS and inflammation induce the activation of programmed cell death in brain tissue, giving rise to the pathogenesis of AD [5].

Previously, the inhibitory mechanisms of amyloid-β peptide production and aggregation via BACE1 have been most intensively investigated. However, downstream processes involving neuronal oxidative stress and inflammation are currently gaining attention and have become strategies for the design and development of disease-modifying Alzheimer’s drugs. Compounds with the capacities of both neuroprotection against neurotoxic ROS and anti-inflammation have been deemed to, altogether, be beneficial to promoting synergistic effects to treat AD. As a result, they can increase the potencies of novel AD drug candidates [6,7,8]. Additionally, during the past few decades, several single-targeted AD drugs have failed in clinical trials, whereas multitargeted drugs or multitargeted-single-ligand approaches have become more promising treatments. Diseases with etiological complexity, like AD, could benefit from this new paradigm shift. These novel treatments have also recently attained more acceptance in AD research and clinical practices [9].

For drugs targeting the central nervous system, it is essential that AD drugs possess the capacity to pass the blood–brain barrier (BBB). Prior studies have shown that ascorbic acid crosses the BBB via interaction with sodium-dependent vitamin C transporter-2 (SVCT2) [10]. Additionally, its oxidized form, known as dehydroascorbic acid, permeates the BBB via glucose transporters (GLUT) [10]. Ascorbic acid is a well-known potent antioxidant. It also reduces amyloid oligomerization associated with a progression of AD pathogenesis [11]. Therefore, ascorbic acid was selected as the functional core structure in the design of the present study. This core domain was used as a skeletal structure for subsequent chemical modifications. Despite the advantages mentioned earlier, ascorbic acid has major pharmacological drawbacks such as low bioavailability [12]. Normally, ascorbic acid predominantly exists in an anion form at neutral pH, causing a slow diffusion rate across the plasma membrane. As the oral dose increases, percent bioavailability is shown to be decreased, potentially due to saturated sodium-dependent vitamin C transporter-1 (SVCT1) [13,14]. Moreover, a distribution from the bloodstream to targeted organs relies on active transporters. Plasma ascorbic acid concentration is, therefore, higher than that in tissue by 2.5-fold [12,15]. As a result, applying a higher daily dose may not be able to overcome this challenge. Moreover, a high intake increases risks of hemolysis in patients with glucose 6 phosphate deficiency, paroxysmal nocturnal hemoglobinuria, oxalate kidney stone development, and iron overload [16,17]. In addition, ascorbic acid can act as a pro-oxidant due to a very good reducing ability. This behavior can reduce catalytic metal, Cu^2+^ or Fe^3+^, to Cu^+^ and Fe^2+^ and generate more ROS [18,19,20].

**Figure 1:**
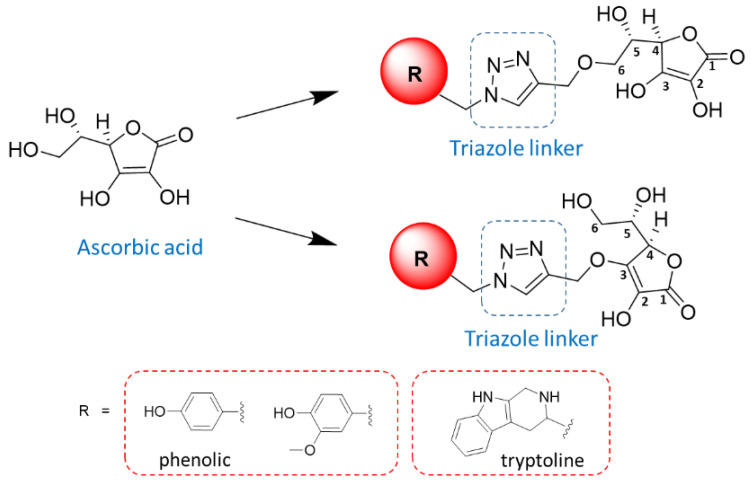
Chemical structure of ascorbic acid and its conjugation via triazole linker with tryptoline and phenolic moieties to create novel compounds with multitargeted functionality to treat Alzheimer’s disease.

The present study aims to construct compounds exerting multitargeted functionality to treat AD. The designs of the novel compounds emphasize the conjugation of the ascorbic core structure to other functional motifs, namely, tryptoline and phenolic moieties, as shown in Figure 1. Tryptoline was selected based on its well-known characteristics as an inhibitor of several enzymes, including beta-secretase (BACE1), cholinesterase (ChE), and monoamine oxidase (MAO) [21,22,23], while the phenolic motif was selected for its well-recognized functioning against oxidative stress and β-amyloid aggregation activities [24,25]. The functional motifs were linked to the ascorbic domain by a triazole ring to generate ascorbic derivatives, which were then investigated for inhibition against BACE1, amyloid aggregation, oxidative stress, and inflammation. In silico predictions of the binding capacities between the newly synthesized ascorbic derivatives to SVCT2, targeting BBB permeation, were also evaluated.

## 2. Results and Discussion

### 2.1. Synthesis

**Figure 2:**
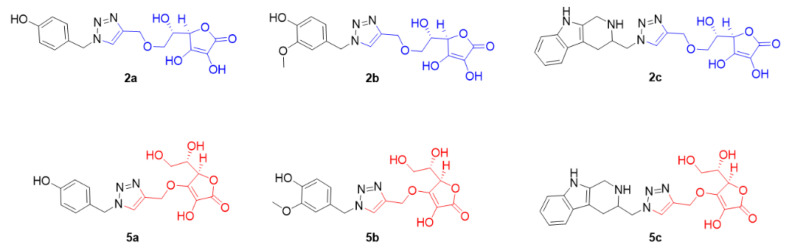
Structures of multifunctional novel ascorbic derivatives.

Six ascorbic derivatives (Figure 2) were designed as novel compounds, with multifunctional activities impeding amyloid cascade toxicity pathways. In the first series, ascorbic acid was substituted at hydroxyl position C-6 to generate derivatives **2a**–**2c**. In the second series, ascorbic acid was substituted at hydroxyl position C-3 to generate derivatives **5a**–**5c**. The syntheses of these derivatives were carried out by the conjugating reactions of the ascorbic acid core structure with either tryptoline or phenolic groups via the triazole linker. The yields of these compounds ranged between 8.58% and 24.61%. The compounds’ yields (Table S1) and NMR spectrums (Figures S1–S12) are reported in the supplementary information. The derivatives’ multifunctional modes of activities were determined in the present study by in vitro chemical-based assays, cell-based assays, and in silico assays.

### 2.2. Biological Activity Assays

Ascorbic acid derivatives **2a**–**2c** and **5a**–**5c** were evaluated for biological activities, including BACE1 inhibition, amyloid aggregation inhibition, and antioxidant, neuroprotection, and anti-inflammation activities. Table 1 shows the results for BACE1 inhibition, amyloid aggregation inhibition, and antioxidant activities at a concentration of 400 μM. Compounds **2c** and **5c** possessed moderate BACE1 inhibitory activities with %inhibition of 23.49 and 27.33, respectively. The BACE1 inhibitory activities of **2c** and **5c** were less than BACE1 inhibitor I (%inhibition of 99.14 at a concentration of 100 μM). However, these two compounds possessed inhibitory activity against BACE1 at least three times higher than that of the ascorbic acid core structure (%inhibition of 7.84). The IC_50_ value of compounds **2c** and **5c** were 725.70 and 593.10 µM, respectively. This suggests that the BACE1 inhibitory activities of the core structure were improved by the conjugation to the tryptoline motif.

**Figure 3:**
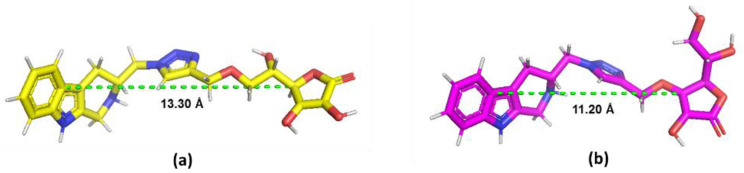
Distance between the aromatic end group and the ascorbic furanone ring in the 3D structure of ascorbic derivatives: (**a**) **2c** (yellow) and (**b**) **5c** (magenta).

All ascorbic derivatives exhibited the potential for amyloid aggregation inhibition in the range of 19.58–71.79% and oxidative stress inhibition in the range of 62.81–94.67%. These findings were expected due to the previously reported characteristics of the ascorbic core structure. Despite lower inhibitory activities than curcumin against amyloid aggregation (94.45% inhibition at a concentration of 100 μM), the newly synthesized compounds **2c** and **5c** were able to hamper amyloid aggregation in a similar inhibitory magnitude to ascorbic acid at a concentration of 400 μM. Compounds **2c** and **5c**, containing the tryptoline motif, were the best lead compounds for the inhibition of amyloid aggregation, with IC_50_ values of 92.33 and 136.00 µM, respectively, which were stronger than that of ascorbic acid (IC_50_ value of 146.10 µM). This suggests that the tryptoline moiety, conjugated to the core structure, contributes to the rise of the amyloid aggregation inhibition activities of compounds **2c** and **5c**. The substitution of the hydroxyl group at the C-6 position of the ascorbic core structure led to higher antiamyloid aggregation activity than that of substitution at the C-3 position. Additionally, the 3D structural model of compound **2c** showed that the distance between the aromatic end group and the ascorbic furanone ring was 13.3 Å (Figure 3A). This distance is in good agreement with the previously reported length of 13–14 Å between the aromatic nucleus of the amyloid aggregation inhibitor [26]. On the contrary, for compound **5c**, the distance was 11.2 Å (Figure 3B), which was not within the optimal range. The differences of this crucial distance might be the explanation for the higher inhibitory activity of compound **2c** against amyloid aggregation than that of compound **5c**.

Compounds **2c** and **5c** were the strongest antioxidants among the derivatives, with IC_50_ values of 72.26 and 62.89 µM, respectively. However, they both exhibited lower antioxidant potential than ascorbic acid (IC_50_ value of 21.28 µM) by 3.0- to 3.4-fold, potentially due to the structure′s steric hindrance.

The results from the in vitro assays in the present study led to the conclusion that the tryptoline moiety attributed to the multifunctional activities of compounds **2c** and **5c**. With regard to possession of BACE1 inhibition, amyloid aggregation inhibition, and antioxidant activities, the novel compounds **2c** and **5c** could potentially produce the mentioned synergistic effects for AD treatment.

Neuroprotective activities against oxidative stress of all the derivatives (**2a**–**2c** and **5a**–**5c**) and ascorbic acid were evaluated using cell viability assays of P19 embryonal carcinoma cells. The P19 embryonal carcinoma cell is a pluripotent stem cell line that can be differentiated into cholinergic neurons by all transretinoic acids [27,28,29]. The P19-derived neurons exhibit many characteristics of mature CNS neurons, containing neurotransmitter acetylcholine [28], which can be used as a model to evaluate neurotoxicity and neuroprotective activity [29,30,31,32,33]. In the present study, neurotoxicity assays were performed prior to neuroprotective activity assays, with test compounds at various concentrations of 1 to 10^4^ nM to evaluate nontoxic concentrations. Cell viability was measured by the XTT assay method, and 0.5% DMSO was used as a control. Ascorbic derivatives at 1 nM did not exhibit cytotoxicity to P19-derived neurons (see supplementary information, Figure S13). Conversely, percent cell viability was shown to be decreased by ascorbic acid. This could partly be explained by ROS generation via an intracellular ascorbate-mediated production of H_2_O_2_ [34]. However, this effect depends on cell type and growth conditions [35]. Noticeably, the ascorbic derivatives exhibited higher percent cell viability than those in ascorbic acid and 0.5% DMSO. This implies that a structural modification of ascorbic acid might help reduce pro-oxidation activity by potentially reducing reactivity or increasing steric hindrance of the structure [36,37]. However, further experiments are required to assess intracellular ROS levels upon exposure to these derivatives. 

**Figure 4:**
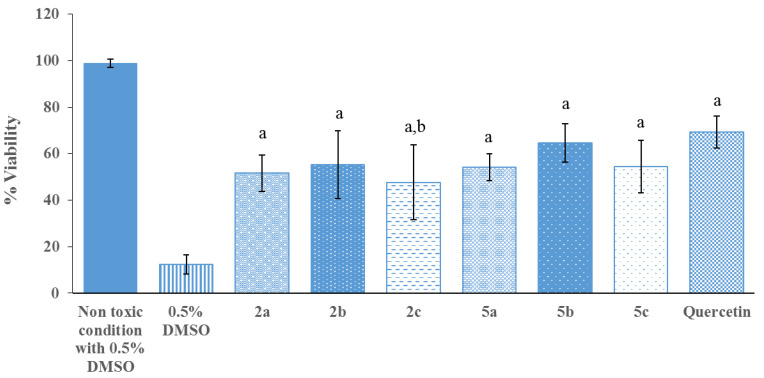
Neuroprotective activities of ascorbic derivatives at a concentration of 1 nM on cultured P19-derived neurons evaluated by serum-deprivation-induced oxidative stress. α-MEM, supplemented with 10 μM Ara-C, and 1% antibiotics–antimycotic solution without FBS were used to induce the oxidative stress condition. Each bar is presented as mean ± SD (*n* = 3). Letter a denotes a significant difference compared with the toxic condition (*p* < 0.05), and letter b denotes a significant difference compared with quercetin (1 nM) positive control (*p* < 0.05), based on one-way ANOVA using the Origin Pro 9.0 program, Northampton, MA, USA.

The ascorbic derivatives (**2a**–**2c** and **5a**–**5c**), at a nontoxic concentration (1 nM), were selected to be further characterized for their neuroprotective effects against the toxic conditions induced by serum deprivation. Serum is necessary for cell growth in cell culture systems because it contains essential growth elements. Therefore, when cultured cells are exposed to serum-deprivation conditions, this induces oxidative stress, leading to apoptosis [38]; this can be used as an in vitro model to assess neuroprotective activity. All ascorbic derivatives (**2a**–**2c** and **5a**–**5c**) showed significantly higher percentages of cell survival than that of 0.5% DMSO in the toxic conditions, which was the absence of fetal bovine serum (FBS; Figure 4; *p* < 0.05). Moreover, there were no statistical differences in neuroprotective activities among compounds **2a**, **2b**, **5a**–**5c**, and quercetin (positive control; *p* > 0.05). However, because **2a** and **5c** (1 nM) showed significantly higher viability than 0.5% DMSO under a nontoxic condition (Figure S13), the observed neuroprotective activities may be caused by this underlying effect. This effect could be partly due to increased cell numbers or mitochondrial activities since the XTT assay is generally used to measure cellular metabolic activities as the indicators of cell viability, cytotoxicity, and proliferation [39,40,41]. Ascorbic acid and phenolic group have been previously shown to increase cell numbers [42,43,44,45] and cellular ATP production [43,46]. Additionally, tryptoline derivatives and phenolic derivatives were also able to promote neurite outgrowth [47], which may be associated with increased mitochondria numbers, accumulated within the neurite [48]. To understand the potential mechanisms of increased percent cell viability of compounds **2a** and **5c** (1 nM) under a nontoxic condition, cell proliferation and intracellular ATP levels should be further investigated.

The in vitro antioxidant activities of the synthesized compounds were in the micromolar range for the chemical-based assays. However, the neuroprotective effects against neurotoxic ROS of these compounds in the cell-based assays were in the nanomolar range. The differences in concentration magnitude might be partly explained by the experimental conditions. There are several cellular defense mechanisms against ROS in the cell-based study models, including mediation via enzymatic and nonenzymatic reactions. Therefore, the effects of ascorbic acid and its derivatives against oxidative stress and cell apoptosis have been determined by diverse markers at the cellular level. These include reduction of malondialdehyde (MDA) [49,50], upregulation of the BCL2 gene (antiapoptotic regulator), and downregulation of the TP53 gene (proapoptotic factor) [51].

Collectively, the results presented in this study prompted us to form conclusive assumptions that compounds **2c** and **5c**, rather than other derivatives, had the best potential for multifunctional BACE1 inhibition, amyloid aggregation inhibition, antioxidant, and neuroprotection activities. Thus, these two compounds were designated as the lead compounds selected to be further investigated for anti-inflammatory activities at the cellular and molecular levels. The ascorbic acid core structure and a well-known anti-inflammatory drug, indomethacin, were also included in the studies for comparison to the lead compounds.

Cyclooxygenase-2 (*COX*-2) and inducible nitric oxide synthase (*iNOS*) were appointed as the inflammatory marker genes and study models for anti-inflammatory activities of the lead compounds. These two genes were previously reported to be linked to AD. *COX*-2 expression is markedly elevated in the AD brain [6]. Regulation of the *COX*-2 gene has also been correlated with clinical dementia in AD patients [52]. Additionally, the deficiency of *iNOS* gene expression has been proven to substantially protect AD-like mice from premature mortality, cerebral plaque formation, and increased β-amyloid levels. Therefore, *iNOS* may play a crucial role in driving an initiation of β-amyloid deposition and disease progression [7].

The cell-based studies were designed to profile differential expressions of *COX*-2 and *iNOS* genes in comparison with 0.001% DMSO control by using lipopolysaccharide (LPS)-activated RAW 264.7 macrophages. The effects of ascorbic acid, **2c**, and **5c** on the viability of RAW 264.7 cells were determined by MTT assays. The results suggest that all compounds, at concentrations from 0.001 to 100 nM, did not exhibit cytotoxicity to RAW 264.7 after 24-h exposure. Cell viabilities were significantly higher than 80% in all experiments (*n* = 3, *p*-value < 0.05; see supplementary information, Figure S14).

**Figure 5:**
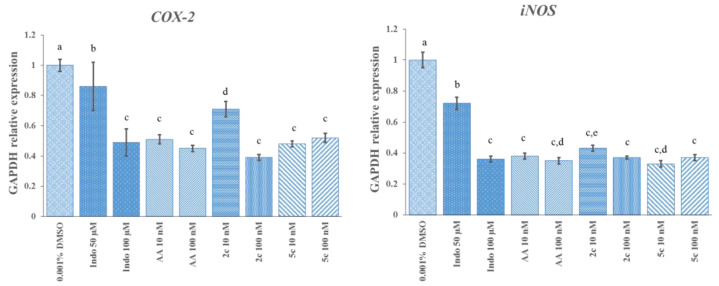
Effects of ascorbic acid, compound **2c**, and compound **5c** at 10 and 100 nM, on mRNA expression levels of *COX*-2 (cyclooxygenase-2) and *iNOS* (inducible nitric oxide synthase) genes in LPS inflammatory-activated RAW 264.7 macrophages. Each bar is presented as mean ± SD (*n* = 3). Different letters (a, b, c, d, and e) indicate significant differences of means between the groups (*p*-value < 0.05) based on Tukey’s HSD one-way ANOVA using PAST version 3.14, Oslo, Norway.

The expression levels of the two inflammatory marker genes were measured by two-step reverse transcription-quantitative PCR (RT-qPCR), with GAPDH as the reference gene. RAW 264.7 cells were pretreated with either ascorbic acid, **2c**, or **5c** at two different concentrations (10 and 100 nM) for one hour prior to induction of the inflammatory process by LPS. Figure 5 shows that ascorbic acid, **2c**, and **5c** were able to downregulate the expression of both *COX*-2 and *iNOS* genes compared to 0.001% DMSO control (*n* = 3, *p*-value < 0.05). Additionally, the magnitudes of the downregulation of both genes by **2c** and **5c** were notably stronger than that of indomethacin (50 µM) by about 1.5- to 2-fold.

The gene expression studies enabled us to effectively assess a pattern of the anti-inflammatory capacities of ascorbic acid, **2c**, and **5c** at the molecular level. The expression of *COX*-2 decreased between 30% to 70%, whereas *iNOS* expression was suppressed by about 50% to 70% by ascorbic acid, **2c**, and **5c**. The ability to inhibit *COX*-2 gene expression was ranked from high to low, as follows: **2c** (100 nM), **5c** (10 and 100 nM), ascorbic acid (10 and 100 nM), indomethacin (100 µM); **2c** (10 nM), and indomethacin (50 µM). Additionally, the capacity for *iNOS* inhibition was ranked from high to low, as follows: **5c** (10 nM), ascorbic acid (100 nM); **2c** (100 nM), **5c** (100 nM), ascorbic acid (10 nM), indomethacin (100 µM), **2c** (10 nM), and indomethacin (50 µM). This leads to an initial conclusion that the underlying molecular mechanisms of neuroprotective activities of the lead compounds **2c** and **5c** involve anti-inflammatory activities mediated through the inhibitions of both *COX*-2 and *iNOS* gene expressions. The present study also reaffirms previous studies that have reported the modulation activities of ascorbic acid to downregulate the expressions of *COX*-2 and *iNOS* [53,54]. However, this study is the first to report the inhibitions of *COX*-2 and *iNOS* gene expression by ascorbyl tryptoline derivatives.

### 2.3. In Silico Study of Sodium-Dependent Vitamin C Transporter2 (SVCT2) Binding

Ascorbic acid was selected as a core structure in the present study not only for its multifunctional activities but also its ability to pass through the BBB by binding to an active transporter. Sodium-dependent vitamin C transporter 2 (SVCT2), which is ubiquitously expressed in various brain tissues, including the cortex, cerebellar stem cells, neurons, and neuroblastoma cells, is a well-known transporter of ascorbic acid into the brain [55]. The conjugation of ascorbic acid to several drugs such as nipecotic, kynurenic, and diclophenamic acids and ibuprofen has demonstrated competitive binding with the SVCT2 receptor and increased drug penetration into the brain over nonconjugated drugs [56,57]. Thus, **2c** and **5c** were studied for their binding affinity with the SVCT2 receptor using molecular docking. 

**Figure 6:**
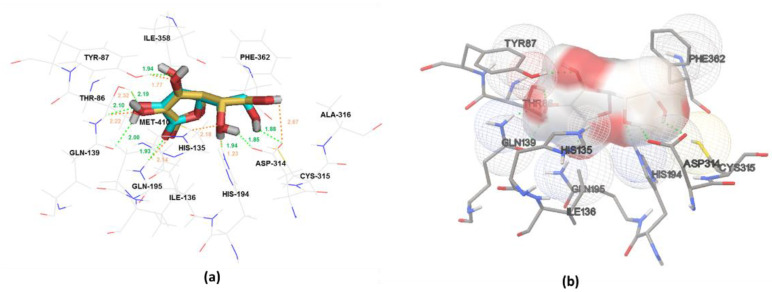
Ascorbic binding modes with sodium-dependent vitamin C transporter-2 (SVCT2) (pdb code: 4RP9). (**a**) Superimposition of ascorbic acid crystal pose (yellow) and dock pose (cyan); (**b**) van der Waals interaction of the residues around the active sites, with ascorbic acid dock pose.

Docking parameters used in the present study were validated by the redocking of ascorbic acid. The docking pose of ascorbic acid was able to bind with the SVCT2 receptor (pdb code: 4RP9) [58], similar to the crystal pose, with the refRMS value of 0.93 Å at the binding free energy of −7.59 kcal/mol. Docking pose and crystal pose both had hydrogen bond interaction with the same amino acids, including Thr86, Tyr87, Gln139, His135, His194, Gln195, and Asp314. Residues having the van der Waals interaction with ascorbic acid around the active site were Thr86, Tyr87, His135, Ile136, Gln139, His194, Gln195, Asp314, Cys315, Ala316, Ile358, Phe362, and Met410. The binding modes of ascorbic acid and the SVCT2 transporter are shown in Figure 6.

*In silico* prediction by a docking study revealed that the designed compounds, **2c** and **5c,** were able to bind with the SVCT2 receptor in a similar binding mode as ascorbic acid. Compounds **2c** and **5c** exhibited binding free energy values of −10.36 and −11.97 kcal/mol, respectively, which were lower than that of ascorbic acid. This might indicate that compounds **2c** and **5c** were able to form the complex to the transporter with more stable conformations than that of ascorbic acid.

**Figure 7:**
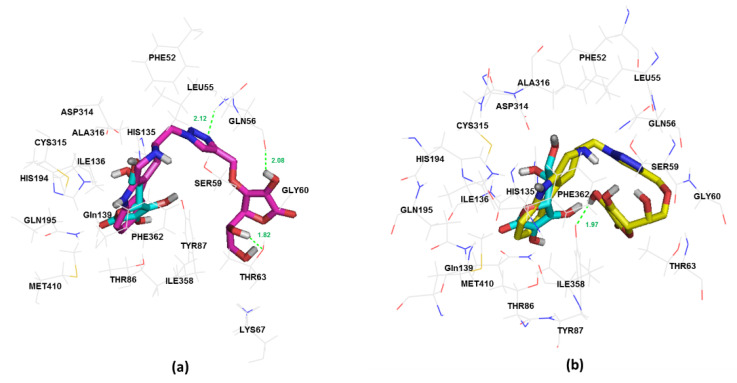
Binding modes of ascorbic derivatives and vitamin C transporter 2 (pdb code: 4RP9). (**a**) Binding position of **5c** (magenta) compared with ascorbic acid (cyan), and (**b**) binding position of **2c** (yellow) compared with ascorbic acid (cyan).

Compound **5c** was able to bind with SVCT2, with a binding energy of -10.36 kcal/mol, and the binding forces were comprised of van der Waals and H-bond interactions of the residues around the active sites. Several amino acids exhibited involvement in the binding interactions at the active sites, including Phe52, Leu55, Gln56, Ser59, Gly60, Thr63, Lys67, Thr86, Tyr87, His135, Ile136, Gln139, His194, Gln195, Asp314, Cys315, Ala316, Ile358, Phe362, and Met410. Compound **5c** formed two hydrogen bonds with Gln56 and Thr63 residues of the transporter (Figure 7a). Nevertheless, compound **2c** showed a binding energy of -11.97 kcal/mol to SVCT2, suggesting a greater binding affinity than that of **5c** despite the similar manner of binding interactions. The explanation might be that compound **2c** interacts differently with SVCT2 at the residue positions of Lys67 and Tyr87 (Figure 7b).

Interestingly, compounds **2c** and **5c** are bound to the transporter at the ascorbic position via the tryptoline moiety instead of the ascorbic acid core structure. However, both **2c** and **5c** were able to bind with the transporter at the amino acid residues, similar to the ascorbic acid binding site (Thr86, Tyr87, His135, Ile136, Gln139, His194, Gln195, Asp314, Cys315, Ala316, Ile358, Phe362, and Met410), a cocrystallized ligand. Thus, the computational analysis presented in this study indicates that both **2c** and **5c** utilize sodium-dependent vitamin C transporter 2 (SVCT2) to facilitate permeability across the BBB into the brain.

## 3. Conclusions

The most potent lead compounds in the present study are **2c** and **5c**. Both novel compounds showed promising multifunctional activities against amyloid cascade, including BACE1 inhibition, amyloid aggregation inhibition, antioxidant activity, neuroprotective activity, and anti-inflammatory activity. Chemical-based assays showed that BACE1 inhibition, amyloid aggregation inhibition, and antioxidant activity were exerted in the micromolar range for both **2c** and **5c**. However, the neuroprotective and anti-inflammatory activities, determined by cell-based assays, were present at the nanomolar level for both compounds. The imbalance of activities on different biological targets indicates that **2c** and **5c** should be used for anti-inflammation and neuroprotection; however, the lower potency of BACE1 inhibition and anti-amyloid aggregation might help by increasing the synergistic activity of the compounds. Moreover, the in silico study predicted that **2c** and **5c** could potentially pass through the BBB into the brain via interaction with the transporter SVCT2. Compounds **2c** and **5c** are potential novel candidates to be further investigated in preclinical studies. However, brain transport of these newly synthesized compounds should be further confirmed in animal studies. Additionally, the activities of these compounds are still required to be improved. The target site of actions, particularly in brain cells, and functional potency are essential to investigate. Regarding the low bioavailability of ascorbic acid, compounds **2c** and **5c** are also required to be further assessed for their pharmacokinetic profiles. The molecular structures of **2c** and **5c** could also be further modified and synthesized as prodrugs to enhance and extend their capacities to treat AD beyond their structural breakdown, as a result of drug metabolism, which may produce active metabolites such as ascorbic acid (a potent amyloid aggregation inhibitor and antioxidant and anti-inflammatory agent) and tryptoline (BACE1, ChE, and MAO inhibitors [21,22,23]). In conclusion, the novel compounds **2c** and **5c** are potential candidates in the discovery and development of new multitargeted AD drug designs.

## 4. Materials and Methods

### 4.1. General

Chemical reagents were purchased from Aldrich, Chem-Impex, or TCI chemical. ^1^H-NMR and ^13^C-NMR spectra were acquired on Bruker Avance 300 or 400 MHz instruments. Mass spectra were recorded on LCMS Bruker MicroTof. IR spectra were recorded on Nicolet FTIR 550. BACE1 enzyme and BACE1 substrate were purchased from Sino Biological^®^, Beijing, China and Calbiochem^®^, San Diego, CA, USA, respectively. Amyloid-β (1–42) from Anaspec^®^, Fremont, CA, USA was used in ThT assays. Neuroprotective assay statistics were determined by ANOVA, calculated with Origin Pro 9.0 program, Northampton, MA, USA. Statistical analysis of cell viability and gene expression data for the anti-inflammatory activity study was conducted by Tukey’s HSD one-way ANOVA using PAST version 3.14, Oslo, Norway. Molecular dockings were performed using AutoDockTools-1.5.6, San Diego, CA, USA. All compounds were generated and optimized with ChemDraw Ultra 13.0 and ChemBio3D Ultra 13.0, Waltham, MA, USA. Microsoft Excel 2013, Redmond, WA, USA and GraphPad Prism version 5.02, San Diego, CA, USA were used in the generation of graph visualization and the calculation of IC_50_ values.

### 4.2. Synthesis

**Scheme 1:**
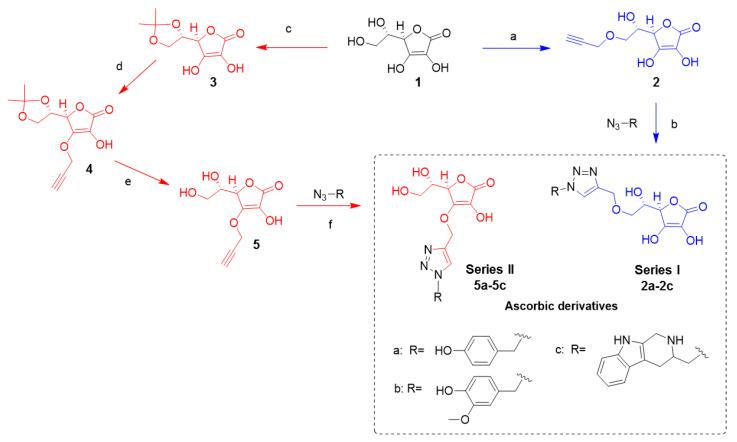
The synthesis route of ascorbic derivatives. (a) K_2_CO_3_, propargyl bromide, DMSO/THF (9:8), rt, 2 h. (b) N_3_R, 5% mol CuSO_4_, 20% mol NaAsc, t-BuOH/EtOH/H_2_O (2:2:1), rt, 2 h. (c) 2,2 dimethoxypropane, p-toluenesulfonyl chloride, rt, 24 h. (d) K_2_CO_3_, propargyl bromide, DMSO/THF (9:8), rt, 2 h. (e) 12 N HCl, THF, rt, 2.5 h. (f) N_3_R, 5% mol CuSO_4_, 20% mol NaAsc, t-BuOH/H_2_O (4:2) or t-BuOH/EtOH/H_2_O (4:4:2), rt, 2 h.

Two series of ascorbic derivatives were synthesized from ascorbic alkyne, **2** and **5**, reacting with azides **a**–**c** by a copper (I)-catalyzed azide–alkyne cycloaddition reaction. Briefly, the hydroxyl group of ascorbic acid **1** at carbon position 6 was substituted with the propargyl group to yield the ascorbic alkynes **2**. Additionally, ascorbic alkynes **5** were synthesized in three steps. Firstly, the hydroxyl group, at carbon position 5 and 6, were protected with the acetonide group to generate compound **3**. Secondly, the hydroxyl group of compound **3**, at carbon position 3, was substituted with the propargyl group to yield compound **4**. Finally, the acetonide protecting group of compound **4** was cleaved in an acidic condition to yield ascorbic alkynes **5**. The complete routes of synthesis are shown in Scheme 1.

#### 4.2.1. (*R*)-3,4-Dihydroxy-5-((S)-1-hydroxy-2-(prop-2-yn-1-yloxy)ethyl)furan-2(5H)-one; **2**

Ascorbic alkyne **2** was synthesized according to the procedure of Wimalasena and Mahindaratne [59]. A mixture of ascorbic acid (2.0000 g, 9.34 mmol) and 0.5 equivalent of K_2_CO_3_ (0.6452 g, 4.67 mmol) in DMSO/THF (9:8) was stirred at room temperature for 20 min. Propargyl bromide (0.83 mL, 9.34 mmol) in the same solvent was added dropwise to the reaction by dropping funnel. The reaction was vigorously stirred at room temperature under nitrogen for 2 h. The reaction was diluted with water (30 mL) and extracted with ethyl acetate (3 × 30 mL). The organic layer was washed with brine and dried over Na_2_SO_4_. The solvent was removed under reduced pressure, and the obtained residue was purified by column chromatography (Hex/EtOAc 2:3) to yield of yellow viscous oil (0.5832 g, 29%); FTIR (ATR, cm^−1^): 3425 (O-H, st), 3279 (O-H, st), 2983, 2957, 2894 (aliphatic C-H, st), 2123 (C≡C, st), 1771 (C=O, st), 1695 (C=C, st), 1326, 1210, 1127 (C-O, st); ^1^H-NMR (300 MHz, DMSO-d_6_): δ 2.47 (1H, m), 2.66 (1H, dd, *J* = 2.70, 16.45 Hz), 2.95 (1H, t, *J* = 2.68 Hz), 3.84 (1H, dd, *J* = 4.22, 9.40 Hz), 4.12 (1H, dd, *J* = 6.18, 9.41 Hz), 4.27 (1H, m), 4.52 (1H, s), 5.60 (1H, d, *J* = 4.14 Hz), 5.96 (1H, s), 6.96 (1H, s); HRMS (ESI) *m*/*z* calcd for [M]^+^ 214.04774, Found 237.03855 [M+Na]^+^.

#### 4.2.2. (*R*)-5-((S)-2,2-Dimethyl-1,3-dioxolan-4-yl)-3,4-dihydroxyfuran-2(5H)-one; **3**

Ascorbic acid (20.0000 g, 113.56 mmol) was dissolved in 80 mL of acetone. Then, 2,2 dimethoxypropane (13.91 mL, 113.56 mmol) and *p*-toluenesulfonyl chloride (43.3000 g, 227.118 mmol) was added to the reaction and stirred at room temperature for 24 h. After the reaction was completed, the reaction mixture was filtered and the precipitate was washed with cooled acetone to yield of white powder (16.1737 g, 65.88%); m.p. 218–220 °C; FTIR (ATR, cm^−1^): 3236 (O-H, st), 2957, 2923, 2853 (aliphatic C-H, st), 1748 (C=O, st), 1658 (C=C, st), 1330, 1137, 1063 (C-O, st); ^1^H-NMR (300 MHz, DMSO-d_6_): 1.24 (6H, s), 3.88 (1H, dd, *J* = 6.32, 8.40 Hz), 4.10 (1H, dd, *J* = 7.05, 8.36 Hz), 4.26 (1H, td, *J*= 2.92, 6.48 Hz), 4.71 (1H, d, *J* = 2.91 Hz), 8.49 (1H, s), 11.31 (1H, s); HRMS (ESI) *m*/*z* calcd for [M]^+^ 216.06339, Found 239.05475 [M+Na]^+^.

#### 4.2.3. (*R*)-5-((S)-2,2-Dimethyl-1,3-dioxolan-4-yl)-3-hydroxy-4-(prop-2-yn-1-yloxy)furan-2(5H)-one; **4**

Compound **4** was synthesized according to the procedure of Wimalasena and Mahindaratne. A mixture of compound **3** (2.0000 g, 9.25 mmol) and 0.5 equivalent of K_2_CO_3_ (0.6392 g, 4.63 mmol) in DMSO/THF (9:8) was stirred at room temperature for 20 min. Propargyl bromide (0.80 mL, 9.25 mmol) in the same solvent was added dropwise to the reaction by dropping funnel. The reaction was vigorously stirred at room temperature under nitrogen for 2 h. The reaction was diluted with water (30 mL) and extracted with ethyl acetate (3 × 30 mL). The organic layer was washed with brine and dried over Na_2_SO_4_. The solvent was removed under reduced pressure and the obtained residue was purified by column chromatography (Hex/EtOAc 8:2) to yield of yellow viscous oil (1.2025 g, 51.13%); FTIR (ATR, cm^−1^): 3285 (O-H, st), 2987, 2937, 2897 (aliphatic C-H, st), 2130 (C≡C, st), 1764 (C=O, st), 1695 (C=C, st), 1373, 1319, 1213, 1143, 1060 (C-O, st); ^1^H-NMR (300 MHz, DMSO-d_6_): 1.26 (6H, s), 3.72 (1H, t, *J* = 2.42 Hz), 3.86 (1H, dd, *J* = 6.04, 8.44 Hz), 4.10 (1H, dd, *J* = 7.09, 8.38 Hz), 4.23 (1H, m), 4.84 (1H, d, *J* = 2.81 Hz), 5.04 (1H, m), 9.27 (1H, s); HRMS (ESI) *m*/*z* calcd for [M]^+^ 254.07904, Found 256.26494 [M+2H]^+^, 277.06949 [M+Na]^+^.

#### 4.2.4. (*R*)-5-((S)-1,2-Dihydroxyethyl)-3-hydroxy-4-(prop-2-ynyloxy)furan-2(5H)-one; **5**

Compound **5** was synthesized by deprotection of the acetonide group in an acidic condition. The pH of the compound **4** solution (0.5923 g, 2.33 mmol) in THF was adjusted to pH 1 by 1 mL of 12 N HCl. The resulting mixture was stirred at room temperature for 2.5 h. After reaction was completed, the reaction mixture was diluted with water (30 mL) and extracted with ethyl acetate (3 × 30 mL). The organic layer was washed with brine and dried over Na_2_SO_4_. The solvent was removed under reduced pressure to obtained yellow oil (0.4298 g, 86.14%). This residue was purified by column chromatography (Hex/EtOAc/MeOH 4:6:0.1) for identification; FTIR (ATR, cm^−1^): 3360, 3290 (O-H, st), 2923, 2853 (aliphatic C-H, st), 2123 (C≡C, st), 1759 (C=O, st), 1696 (C=C, st), 1327, 1156, 1064 (C-O, st); ^1^H-NMR (300 MHz, DMSO-d_6_): 3.45 (2H, m), 3.65 (1H, t, *J* = 7.25 Hz), 3.70 (1H, t, *J* = 2.40 Hz), 4.79 (1H, d, *J* = 1.25 Hz), 4.97 (1H, dd, *J* = 15.5, 32.44 Hz), 4.97 (1H, br), 5.11 (1H, dd, *J* = 15.53, 2.41 Hz), 9.07 (1H, s); HRMS (ESI) *m*/*z* calcd for [M]^+^ 214.04774, Found 215.05676 [M^+^H]^+^.

#### 4.2.5. 4-(Azidomethyl)phenol; **a**

4-Hydroxybenzyl alcohol (2.5000 g, 20.00 mmol) and potassium fluoride (1.3922 g, 24.00 mmol) were dissolved in acetic acid (5 mL). The reaction mixture was stirred at 80 °C for 1 h. After reaction was completed, water (30 mL) was added to the reaction mixture and extracted with ethyl acetate (3 × 30 mL). The organic phase was washed with saturated NaHCO_3_, brine, dried over Na_2_SO_4_, and concentrated under reduced pressure. The obtained residue was purified by column chromatography using dichloromethane/ethyl acetate (1:1) to give a 63.13% yield of the intermediate. This intermediate and NaN_3_ (2 eq) were dissolved in dried DMF under nitrogen. Boron trifluoride etherate (3 eq) was added to the reaction and stirred at room temperature, with light protection, for 3 h. After reaction was completed, dichloromethane (30 mL) was added to the reaction and washed with saturated NaHCO_3_, brine, dried over Na_2_SO_4_, and concentrated under reduced pressure. The product was purified by column chromatography (Hex/EtOAc 9:1) to yield of dark liquid (0.4815 g, 16.15%); FTIR (ATR, cm^−1^): 3354 (O-H, st), 3059 (aromatic C-H, st), 2926, 2854 (aliphatic C-H, st), 2097 (N=N=N, st), 1515 (C=C, st), 1264 (C-O, st), 1240 (C-N, st); ^1^H-NMR (300 MHz, DMSO-d_6_): δ 4.29 (2H, s), 6.77 (2H, d, *J* = 8.52 Hz), 7.18 (2H, d, *J* = 8.52 Hz), 9.57 (1H, s). HRMS (ESI) *m*/*z* calcd for [M]^+^ 149.05891, Found 107.05055 [M-42]^+^.

#### 4.2.6. 4-(Azidomethyl)-2-methoxyphenol; **b**

4-Hydroxy-3-methoxybenzyl alcohol (3.0000 g, 19.46 mmol) and potassium fluoride (1.3547 g, 23.35 mmol) were dissolved in acetic acid (5 mL). The reaction mixture was stirred at 80 °C for 1 h. After reaction was completed, water (30 mL) was added to the reaction mixture and extracted with ethyl acetate (3 × 30 mL). The organic phase was washed with saturated NaHCO_3_, brine, dried over Na_2_SO_4_, and concentrated under reduced pressure. The product was purified by column chromatography using dichloromethane to give a 73.10% yield of the intermediate. This intermediate and NaN_3_ (4 eq) were dissolved in dried DMF under nitrogen. Boron trifluoride etherate (6 eq) was added to the reaction and stirred at room temperature with light protection for 3 h. After reaction was completed, dichloromethane (30 mL) was added to the reaction and washed with saturated NaHCO_3_, brine, dried over Na_2_SO_4_, and concentrated under reduced pressure, and the obtained residue was purified by column chromatography using hexane: ethyl acetate (9:1) to yield of yellow liquid (0.4709 g, 13.51%); FTIR (ATR, cm^−1^): 3511, 3423 (O-H, st), 3011 (aromatic C-H, st), 2936, 2844 (aliphatic C-H, st), 2096 (N=N=N, st), 1514 (C=C, st), 1272, 1237 (C-O, st), 1154 (C-N, st); ^1^H-NMR (300 MHz, DMSO-d_6_): δ 3.77 (3H, s), 4.30 (2H, s), 6.78 (2H, d, *J* = 1.07 Hz), 6.94 (1H, s), 9.12 (1H, s). HRMS (ESI) *m*/*z* calcd for [M]^+^ 179.06948, Found 137.06065 [M-42]^+^. 

#### 4.2.7. 4-(Azidomethyl)-2-methoxyphenol; **c**

**Scheme 2:**
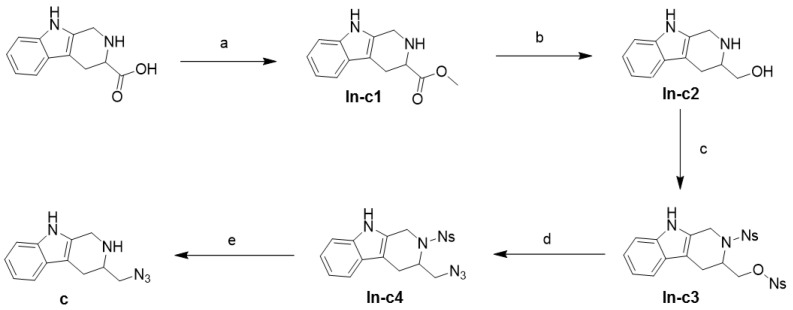
The synthesis route of (3-azidomethyl)tryptoline, **c**. (a) MeOH, H_2_SO_4_, reflux, 18 h. (b) NaBH_4_, MeOH/THF (1:1), reflux, 18 h. (c) NsCl, TEA, CH_2_Cl_2_, rt, 4 h. (d) NaN_3_, DMF, 70 °C, 6 h. (e) *p*-toluenethiol, DMF, 50 °C, 2 h.

Azide compound **c** was synthesized, following the previous method of tryptoline azide, with some modification of deprotection reagent [60]. Briefly, the carboxylic group of tryptoline-3-carboxylic acid was firstly activated by esterification to obtain **In-c1**, which was further reduced to yield **In-c2**. Then, the hydroxyl group of **In-c2** was replaced with nosyl, Ns, a good leaving group for azide substitution. Finally, the nosyl group at amine, Ns, was removed by *p*-toluenethiol in the basic condition to yield (3-azidomethyl)tryptoline **c**, as shown in Scheme 2.

#### 4.2.8. Methyl 2,3,4,9-tetrahydro-1H-pyrido [3,4-b]indole-3-carboxylate; **In-c1**

A mixture of tryptoline-3-carboxylic acid (5.00 g, 23.10 mmol) and 3 mL of sulfuric acid in 30 mL of methanol was heated at reflux for 18 h. Then, 30 mL of water was added to the reaction mixture, and the organic solvent was removed under reduced pressure. After pH was adjusted to pH 8 with saturated sodium bicarbonate solution, the aqueous phase was extracted with ethyl acetate (30 mL × 2). The organic solution was dried over sodium sulfate, concentrated, and purified by crystallization with ethyl ether and methanol (10:2) to yield compound **In-c1**. Product was a light brown crystal (5.2997 g, 99.64%); m.p. 166–168 °C; FTIR (ATR) (cm^−1^): 3388, 3299 (N-H, st), 3056 (aromatic C-H, st), 2950, 2845 (aliphatic C-H, st) 1732 (C=O, st), 1452, 1436 (aromatic C=C, st), 1331 (C-N, st), 1269 (C-O, st), 1201 (C-N, st), 741 (aromatic C-H, bending); ^1^H-NMR (300 MHz, DMSO-d_6_): δ 10.76 (1H, s), 7.39 (1H, d, *J* = 7.6 Hz), 7.28 (1H, d, *J* = 7.9 Hz), 7.03 (1H, t, *J* = 6.9 Hz), 6.95 (1H, t, *J* = 6.8 Hz), 4.02 (2H, q, *J* = 16.0 Hz), 3.85 (1H, dd, *J* = 8.9, 4.9 Hz), 3.70 (3H, s), 3.45 (1H, s), 2.98 (1H, dd, *J* = 15.0, 4.8 Hz), 2.78 (1H, dd, *J* = 15.1, 8.9 Hz).

#### 4.2.9. (2,3,4,9-Tetrahydro-1H-pyrido [3,4-b]indol-3-yl)methanol; **In-c2**

Compound **In-c1** (5.2997 g, 23.02 mmol), in 30 mL of dried methanol and dried tetrahydrofuran (1:1), was added slowly to a stirred solution of sodium borohydride (3.0475 g, 50.56 mmol) at room temperature. The mixture was heated to boiling under reflux for 18 h. The solution was evaporated under reduced pressure to remove organic solvent. Then, 30 mL water was added and extracted with ethyl acetate (30 mL × 2). The organic solution was dried, concentrated, and purified by column chromatography on silica gel (CH_2_Cl_2_/EtOAc/MeOH/NH_4_OH 5:4.5:0.5:0.01) to yield compound **In-c2**. The product was white powder (2.7148 g, 58.32%); m.p. 194–196 °C; FTIR (ATR) (cm^−1^): 3395 (indole N-H, st), 3279 (N-H, st), 3200 (O-H, st), 3050 (aromatic C-H, st), 2885, 2841 (aliphatic C-H, st), 1450 (aromatic C=C, st), 1326 (C-N, st), 1215 (C-O, st), 1023 (C-N, st) 743 (aromatic C-H, bending); ^1^H-NMR (300 MHz, DMSO-d_6_): δ 10.65 (1H, s), 7.33 (1H, d, *J* = 7.5 Hz), 7.25 (1H, d, *J* = 7.8 Hz), 6.99 (1H, t, *J* = 6.8 Hz), 6.92 (1H, t, *J* = 6.8 Hz), 4.69 (1H, t, *J* = 5.4 Hz), 3.91 (1H, s), 3.59–3.41 (1H, m), 2.85 (1H, dd, *J* = 10.2, 5.4 Hz), 2.62 (1H, dd, *J* = 14.9, 4.0 Hz), 2.37–2.22 (2H, m).

#### 4.2.10. (*S*)-3-(Azidomethyl)-2-(4-nitrophenylsulfonyl)-2,3,4,9-tetrahydro-1H –pyrido[3,4-b]indole; **In-c4**

A mixture of compound **In-c2** (2.0654 g, 10.21 mmol) and triethylamine (2.25 mL, 30.64 mmol) in 30 mL of dried dichloromethane was added with 4-nitrobenzenesulfonyl chloride (5.6580 g, 25.53 mmol) in dried dichloromethane at 0 °C. The reaction mixture was adjusted to room temperature and stirred for 4 h. Water (30 mL) was added to the reaction. Organic phase was washed with 1N HCl, saturated NaHCO_3_ and brine. The organic solution was dried and concentrated to yield compound **In-c3** intermediate. This intermediate was redissolved in dried DMF. NaN_3_ (3.3194 g, 51.06 mmol) was added to the solution and stirred at 70 °C for 6 h. After reaction was completed, 30 mL of water was added to the reaction mixture. The resulting solution was extracted with ethyl acetate (30 mL × 2). The organic phase was washed with saturated NaHCO_3_ and brine. The resulting solution was dried, concentrated, and purified by column chromatography on silica gel with CHCl_3_ to yield a yellow powder of compound **In-c4** (3.2969 g, 78.28%); m.p. 205–207 °C; FTIR (ATR) (cm^−1^): 3389 (indole N-H, st), 3018 (aromatic C-H, st), 2927, 2868 (aliphatic C-H, st), 2101 (N=N=N, st), 1667 (N-H, bending), 1529 (N=O, st), 1454 (aromatic C=C, st), 1349 (N=O, st), 1347 (S=O, st), 1167 (S=O, st), 1094 (C-N, st), 854 (nitroaromatic C-N, st), 760 (aromatic C-H, bending); ^1^H-NMR (300 MHz, DMSO-d_6_): δ 10.84 (1H, s, *J* = 8.1 Hz), 8.29 (2H, d, *J* = 8.9 Hz), 8.10 (2H, d, *J* = 8.9 Hz), 7.28 (2H, t, *J* = 6.9 Hz), 7.03 (1H, t, *J* = 7.5 Hz), 6.91 (1H, t, *J* = 7.4 Hz), 4.87 (1H, d, *J* = 17.4 Hz), 4.69–4.59 (1H, m), 4.46 (1H, d, *J* = 17.5 Hz), 3.50 (1H, dd, *J* = 13.0, 9.7 Hz), 3.39 (1H, dd, *J* = 13.0, 5.4 Hz), 2.70–2.57 (2H, m).

#### 4.2.11. (*S*)-3-(Azidomethyl)-2,3,4,9-tetrahydro-1H-pyrido[3,4-b]indole; **c**

A mixture of compound **In-c4** (3.2969 g, 7.99 mmol), potassium carbonate (3.3143 g, 23.98 mmol), and *p*-toluenethiol (4.9643 g, 39.97 mmol) in 5 mL of DMF was stirred at 50 °C for 2 h. After the reaction was completed, 15 mL of saturated aqueous NH_4_Cl was added. The mixture solution was extracted with ethyl acetate (30 × 2 mL). The organic phase was washed with water, saturated NaHCO_3_ and brine. The resulting solution was dried, concentrated, and purified by column chromatography on silica gel (CHCl_3_/MeOH 10:0.2). A yellow powder product of (3-azidomethyl)tryptoline, **c,** was obtained (0.7484 g, 41.19%); m.p. 179–181 °C; FTIR (ATR) (cm^−1^): 3396 (indole N-H, st), 3161 (N-H, st), 3056 (aromatic C-H, st), 2923, 2842 (aliphatic C-H, st), 2100 (N=N=N, st), 1450 (aromatic C=C, st), 1267 (C-N, st), 742 (aromatic C-H, bending); ^1^HNMR (300 MHz, DMSO-d_6_): δ 10.70 (1H, s, H9), 7.34 (1H, d, *J* = 7.57 Hz, H5), 7.26 (1H, d, *J* = 7.82 Hz, H8), 7.00 (1H, t, *J* = 6.83 Hz, H7), 6.93 (1H, t, *J* = 6.79 Hz, H6), 3.91 (2H, s, H1), 3.44 (2H, dd, *J* = 2.51, 6.19 Hz, H10), 3.03 (1H, m, H3), 2.68 (1H, dd, *J* = 4.11, 14.93 Hz, H4b), 2.36 (1H, dd, *J* = 9.92, 14.96 Hz, H4a); HRMS (ESI) *m*/*z* calcd for [M]^+^ 227.1171, Found 228.12485 [M+H]^+^, 144.08179 [M-84]^+^.

#### 4.2.12. (*R*)-3,4-Dihydroxy-5-((S)-1-hydroxy-2-((1-(4-hydroxybenzyl)-1H-1,2,3-triazol-4-yl)metho-xy)ethyl)furan-2(5H)-one; **2a**

A mixture of compound (0.1806 g, 0.843 mmol), 4-(azidomethyl)phenol, **a,** (0.1509 g, 1.012 mmol), 5% mol CuSO_4_, and 20% mol sodium ascorbate in 8 mL of *t*-BuOH/EtOH/H_2_O (2:2:1) was stirred at room temperature for 2 h. After reaction was completed, water (10 mL) was added to the reaction mixture. The aqueous solution was extracted with ethyl acetate (3 × 20 mL). The organic solution was washed with brine, dried, concentrated, and purified by column chromatography (Hex/EtOAc/MeOH 1:4:0.1). A yellow viscous of compound **2a** was obtained (0.0263 g, 8.58%); FTIR (ATR) (cm^−1^): 3358 (O-H, st), 3194 (aromatic C-H, st), 2955, 2851 (aliphatic C-H, st), 1659, 1632 (C=O, st), 1468 (aromatic C=C, st), 1411 (C-N, st), 1237 (C-O, st), 722 (aromatic C-H, bending); ^1^H-NMR (300 MHz, DMSO-d_6_): δ 3.40 (2H, m, H6), 3.62 (1H, d, *J* = 6.27 Hz, H5), 4.75 (1H, d, *J* = 0.74 Hz, H4), 4.82 (1H, s, OH), 5.01 (1H, d, *J* = 5.92 Hz, OH), 5.47 (4H, m, H7, H10), 6.74 (2H, d, *J* = 8.43 Hz, H13, H15), 7.18 (2H, d, *J* = 8.48 Hz, H12, H16), 8.18 (1H, s, H9), 9.00 (1H, s, OH), 9.56 (1H, s, OH); ^13^C-NMR (300 MHz, DMSO-d_6_): δ 53.07 (C10), 62.19 (C5), 64.03 (C7), 68.86 (C6), 74.96 (C4), 115.90 (C13, C15), 120.29 (C2), 124.84 (C9), 126.50 (C11), 130.13 (C12, C16), 142.90 (C8), 150.45 (C14), 157.85 (C3), 170.80 (C1); HRMS (ESI) *m*/*z* calcd for [M]^+^ 363.10665, Found 386.09639 [M+Na]^+^, 364.11406 [M+H]^+^.

#### 4.2.13. (*R*)-3,4-Dihydroxy-5-((S)-1-hydroxy-2-((1-(4-hydroxy-3-methoxybenzyl)-1H-1,2,3-triazol-4-yl)methoxy)ethyl)furan-2(5H)-one; **2b**

A mixture of compound **2** (0.1403 g, 0.655 mmol), 4-(azidomethyl)-2-methoxyphenol, **b,** (0.1409 g, 0.786 mmol), 5% mol CuSO_4_, and 20% mol sodium ascorbate in 8 mL of *t*-BuOH/EtOH/H_2_O (2:2:1) was stirred at room temperature for 2 h. After reaction was completed, water (10 mL) was added to the reaction mixture. The aqueous solution was extracted with ethyl acetate (3 × 20 mL). The organic solution was washed with brine, dried, concentrated and purified by column chromatography (Hex/EtOAc/MeOH 1:4:0.1). A yellow viscous of compound **2b** was obtained (0.0317 g, 12.30%); FTIR (ATR) (cm^−1^): 3360 (O-H, st), 3195 (aromatic C-H, st), 2923, 2852 (aliphatic C-H, st), 1660, 1632 (C=O, st), 1467 (aromatic C=C, st), 1410 (C-N, st), 1264 (C-O, st), 1132 (C-N, st), 737, 704 (aromatic C-H, bending); ^1^H-NMR (300 MHz, DMSO-d_6_): δ 3.41 (2H, m, H6), 3.61 (1H, dd, *J* = 7.38, 13.74 Hz, H5), 3.74 (3H, s, CH_3_), 4.75 (1H, s, H4), 4.82 (1H, t, *J* = 5.66 Hz, OH), 5.01 (1H, d, *J* = 6.15 Hz, OH), 5.47 (4H, m, H7, H10), 6.74 (2H, d, *J* = 8.29 Hz, H12, H16), 6.98 (H, d, *J* = 4.35 Hz, H15), 8.20 (1H, s, H9), 9.01 (1H, s, OH), 9.13 (1H, s, OH); ^13^C-NMR (300 MHz, DMSO-d_6_): δ 53.39 (CH_3_), 56.08 (C10), 62.18 (C5), 64.02 (C7), 68.87 (C6), 74.97 (C4), 113.01 (C12), 115.98 (C15), 120.29 (C2), 121.47 (C16), 124.88 (C9), 126.90 (C11), 142.89 (C8), 147.09 (C14), 148.08 (C13), 150.44 (C3), 170.79 (C1); HRMS (ESI) *m*/*z* calcd for [M]^+^ 393.11721, Found 416.10439 [M+Na]^+^, 394.12280 [M+H]^+^.

#### 4.2.14. (*R*)-3,4-Dihydroxy-5-((S)-1-hydroxy-2-((1-((2,3,4,9-tetrahydro-1H-pyrido[3,4-b]indol-3-yl)-methyl)-1H-1,2,3-triazol-4-yl)methoxy)ethyl)furan-2(5H)-one; **2c**

A mixture of compound **2** (0.2693 g, 1.257 mmol), 3-(azidomethyl)-2,3,4,9-tetrahydro-1H-pyrido[3,4-b]indole, **c,** (0.1858 g, 0.817 mmol), 5% mol CuSO_4_, and 20% mol sodium ascorbate in 8 mL of *t*-BuOH/EtOH/H_2_O (2:2:1) was stirred at room temperature for 2 h. After reaction was completed, water (10 mL) was added to the reaction mixture. The aqueous solution was extracted with ethyl acetate (3 × 20 mL). The organic solution was washed with brine, dried, concentrated, and purified by column chromatography (EtOAc/EtOH 7:3). A light brown powder of compound **2c** was obtained (0.1361 g, 24.52%); m.p. 172–174 °C; FTIR (ATR) (cm^−1^): 3360 (O-H, st), 3192 (aromatic C-H, st), 2922, 2851 (aliphatic C-H, st), 1659, 1632 (C=O, st), 1467 (aromatic C=C, st), 1409 (C-N, st), 1264 (C-O, st), 738, 703 (aromatic C-H, bending); ^1^H-NMR (300 MHz, DMSO-d_6_): δ 2.42 (1H, dd, *J* = 9.84, 14.86 Hz, H12a) 2.67 (1H, dd, *J* = 3.96, 14.98 Hz, H12b), 3.42 (3H, m, H6, H11), 3.65 (1H, m, H5), 3.91 (2H, q, *J* = 16.57 Hz, H10), 4.55 (2H, m, H21), 4.78 (1H, d, 0.93 Hz, H4), 4.84 (1H, s, OH), 5.03 (1H, s, OH), 5.48 (1H, d, *J* = 12.16 Hz, H7a), 5.57 (1H, d, *J* = 12.18 Hz, H7b), 6.93 (1H, t, *J* = 7.28 Hz, H16), 7.01 (1H, t, *J* = 7.48 Hz, H17), 7.27 (1H, d, *J* = 7.84 Hz, H18), 7.34 (1H, d, *J* = 7.51 Hz, H15), 8.27 (1H, s, H9), 9.08 (1H, s, OH), 10.72 (1H, s, NH-indole); ^13^C-NMR (300 MHz, DMSO-d_6_): δ 25.70 (C12), 42.24 (C21), 53.85 (C11), 54.21 (C10), 62.20 (C5), 64.09 (C7), 68.88 (C6), 74.99 (C4), 106.28 (C13), 111.33 (C18), 117.59 (C2), 118.74 (C15), 120.37 (C16), 120.89 (C17), 125.92 (C14), 127.39 (C9), 134.08 (C20), 136.23 (C19), 142.44 (C8), 150.48 (C3), 170.84 (C1); HRMS (ESI) *m*/*z* calcd for [M]^+^ 441.16483, Found 442.17209 [M+H]^+^.

#### 4.2.15. (*R*)-5-((S)-1,2-Dihydroxyethyl)-3-hydroxy-4-((1-(4-hydroxybenzyl)-1H-1,2,3-triazol-4-yl)-methoxy)furan-2(5H)-one; **5a**

A mixture of compound **5** (0.4298 g, 2.007 mmol), 4-(azidomethyl)phenol, **a,** (0.4490 g, 3.010 mmol), 5% mol CuSO_4_, and 20% mol sodium ascorbate in 6 mL of *t*-BuOH/H_2_O (4:2) was stirred at room temperature for 2 h. After reaction was completed, water (10 mL) was added to the reaction mixture. The aqueous solution was extracted with ethyl acetate (3 × 20 mL). The organic solution was washed with brine, dried, concentrated, and purified by column chromatography (Hex/EtOAc/EtOH 1:4:0.1). A yellow semisolid of compound **5a** was obtained (0.0999 g, 13.70%); FTIR (ATR) (cm^−1^): 3358 (O-H, st), 3197 (aromatic C-H, st), 2921, 2851 (aliphatic C-H, st), 1659, 1632 (C=O, st), 1468 (aromatic C=C, st), 1410 (C-N, st), 1264 (C-O, st), 738, 704 (aromatic C-H, bending); ^1^H-NMR (300 MHz, DMSO-d_6_): δ 3.43 (1H, m, H6), 3.61 (1H, t, *J* = 7.09 Hz, H5), 4.75 (1H, s, H4), 4.83 (1H, t, *J* = 5.58 Hz, OH), 5.02 (1H, d, *J* = 6.11 Hz, OH), 5.41 (1H, d, *J* = 12.16 Hz, H7a), 5.46 (2H, s, H10), 5.52 (1H, d, *J* = 12.14 Hz, H7b), 6.74 (2H, d, *J* = 8.41 Hz, H13, H15), 7.18 (2H, d, *J* = 8.45 Hz, H12, H16), 8.18 (1H, s, H9), 9.01 (1H, s, OH), 9.56 (1H, s, OH); ^13^C-NMR (300 MHz, DMSO-d_6_): δ 53.07 (C10), 62.18 (C7), 64.02 (C6), 68.86 (C5), 74.97 (C4), 115.90 (C13, C15), 120.29 (C2), 124.84 (C9), 126.50 (C11), 130.13 (C12, C16), 142.90 (C3), 150.45 (C8), 157.84 (C14), 170.80 (C1); HRMS (ESI) *m*/*z* calcd for [M]^+^ 363.10665, Found 364.11522 [M+H]^+^, 386.09827 [M+Na]^+^.

#### 4.2.16. (*R*)-5-((S)-1,2-Dihydroxyethyl)-3-hydroxy-4-((1-(4-hydroxy-3-methoxybenzyl)-1H-1,2,3-triazol-4-yl)methoxy)furan-2(5H)-one; **5b**

A mixture of compound **5** (0.2319 g, 1.083 mmol), 4-(azidomethyl)-2-methoxyphenol, **b,** (0.2910 g, 1.624 mmol), 5% mol CuSO_4_, and 20% mol sodium ascorbate in 6 mL of *t*-BuOH/H_2_O (4:2) was stirred at room temperature for 2 h. After reaction was completed, water (10 mL) was added to the reaction mixture. The aqueous solution was extracted with ethyl acetate (3 × 20 mL). The organic solution was washed with brine, dried, concentrated, and purified by column chromatography (Hex/EtOAc/MeOH 1:4:0.1). A light yellow semisolid of compound **5b** was obtained (0.1048 g, 24.61%); FTIR (ATR) (cm^−1^): 3358 (O-H, st), 3195 (aromatic C-H, st), 2922, 2851 (aliphatic C-H, st), 1659, 1632 (C=O, st), 1468 (aromatic C=C, st), 1410 (C-N, st), 1264 (C-O, st), 737, 704 (aromatic C-H, bending); ^1^H-NMR (300 MHz, DMSO-d_6_): δ 3.40 (2H, m, H6), 3.61 (1H, dd, *J* = 7.01, 13.35 Hz, H5), 3.74 (3H, s, CH3), 4.75 (1H, d, *J* = 1.20 Hz, H4), 4.82 (1H, t, *J* = 5.64 Hz, OH), 5.01 (1H, d, *J* = 6.14 Hz, OH), 5.41 (1H, d, *J* = 12.18 Hz, H7a), 5.46 (2H, s, H10), 5.53 (1H, d, *J* = 12.19 Hz, H7b), 6.74 (2H, d, *J* = 7.66 Hz, H12, H16), 6.97 (1H, s, H15), 8.20 (1H, s, H9), 9.01 (1H, s, OH), 9.13 (1H, s, OH); ^13^C-NMR (300 MHz, DMSO-d_6_): δ 53.39 (CH_3_), 56.08 (C10), 62.18 (C7), 64.02 (C6), 68.87 (C5), 74.96 (C4), 113.02 (C12), 115.98 (C2), 120.29 (C15), 212.47 (C16), 124.88 (C9), 126.90 (C11), 142.89 (C3), 147.09 (C8), 148.08 (C14), 150.43 (C13), 170.79 (C1); HRMS (ESI) *m*/*z* calcd for [M]^+^ 393.11721, Found 394.12523 [M+H]^+^, 416.10913 [M+Na]^+^.

#### 4.2.17. (*R*)-5-((S)-1,2-Dihydroxyethyl)-3-hydroxy-4-((1-((2,3,4,9-tetrahydro-1H-pyrido[3,4-b]indol-3-yl)methyl)-1H-1,2,3-triazol-4-yl)methoxy)furan-2(5H)-one; **5c**

A mixture of compound **5** (0.5439 g, 2.540 mmol), 3-(azidomethyl)-2,3,4,9-tetrahydro-1H-pyrido[3,4-b]indole, **c,** (0.3607 g, 1.587 mmol), 5% mol CuSO_4_, and 20% mol sodium ascorbate in 8 mL of *t*-BuOH/EtOH/H_2_O (4:4:2) was stirred at room temperature for 2 h. After reaction was completed, alcohol was evaporated out and water (10 mL) was added to the reaction mixture. The aqueous solution was extracted with ethyl acetate (3 × 30 mL). The organic solution was washed with brine, dried, concentrated, and purified by column chromatography (EtOAc/EtOH 9:1). A brown powder of compound **5c** was obtained (0.1493 g, 13.32%); m.p. 144–146 °C; FTIR (ATR) (cm^−1^): 3358 (O-H, st), 3195 (aromatic C-H, st), 2921, 2851 (aliphatic C-H, st), 1741, 1658, 1632 (C=O, st), 1468 (aromatic C=C, st), 1410 (C-N, st), 1220, 1197 (C-O, st), 738, 703 (aromatic C-H, bending); ^1^H-NMR (300 MHz, DMSO-d_6_): δ 2.54 (1H, m, H12a), 2.77 (1H, d, *J* = 12.11 Hz, H12b), 3.40 (2H, dd, *J* = 7.08, 14.72 Hz, H6), 3.65 (2H, d, *J* = 6.89 Hz, H5, H11), 4.03 (3H, m, H10, OH), 4.62 (3H, d, *J* = 5.89 Hz, H21, OH), 4.78 (1H, d, *J* = 1.01 Hz, H4), 5.48 (1H, d, *J* = 12.19 Hz, H7a), 5.58 (1H, d, *J* = 12.19 Hz, H7b), 5.76 (1H, s, OH), 6.95 (1H, t, *J* = 7.07 Hz, H17), 7.03 (1H, t, *J* = 7.45 Hz, H17), 7.29 (1H, d, *J* = 7.91 Hz, H18), 7.36 (1H, d, *J* = 7.60 Hz, H15), 8.32 (1H, d, *J* = 15.52 Hz, H9), 10.80 (1H, s, NH-indole); ^13^C-NMR (300 MHz, DMSO-d_6_): δ 24.98 (C12), 31.15 (C21), 42.03 (C11), 53.77 (C10), 62.19 (C7), 64.04 (C6), 68.90 (C5), 74.99 (C4), 105.97 (C13), 111.45 (C18), 117.73 (C2), 118.93 (C15), 120.40 (C16), 121.18 (C17), 126.06 (C14), 127.13 (C9), 132.51 (C20), 136.34 (C19), 142.57 (C3), 150.45 (C8), 170.82 (C1); HRMS (ESI) *m*/*z* calcd for [M]^+^ 441.16483, Found 442.17604 [M+H]^+^, 464.15840 [M+Na]^+^.

### 4.3. Biological Activity Assays

#### 4.3.1. β-Secretase (BACE1) Inhibition Assay

All ascorbic derivatives (**2a**–**2c** and **5a**–**5c**), at a concentration of 400 µM, were evaluated for β-secretase inhibitory activities by using the FRET assay. Briefly, the reaction composing 30 µL of 0.01 unit/µL BACE1 enzyme stock solution, 20 µL of 5% DMSO of test compound, 30 µL of sodium acetate buffer (pH 4.5), and 20 µL of 125 µM β-secretase substrate IV (Calbiochem^®^, San Diego, CA, USA) was incubated at 37 °C for 30 min. Fluorescence was then measured at E_x_ = 380 nm and E_m_ = 510 nm using a SpectraMax Gemini EM^™^, San Jose, CA, USA [26,61]. β-secretase inhibitor I (Anaspec^®^, Fremont, CA, USA) was used as a positive control, at a concentration of 100 µM. The experiments were performed in triplicate. For the compounds exerting inhibitory activities more than 20%, an IC50 value was further determined.

#### 4.3.2. Amyloid Aggregation Inhibition Assay

The ascorbic derivatives ware evaluated for activities of amyloid β aggregation inhibition at a concentration of 400 µM. Briefly, in each well of transparent plate, 9 µL of 25 μM working solution of amyloid-β (1-42) peptide and 1 µL of test compound were gently mixed and then incubated in the dark at 37 °C for 48 h. After the incubation, 200 µL of 5 µM ThT (Sigma^®^, Saint Louis, MO, USA) in 50 mM Tris buffer (pH 7.4) was added to each well. Fluorescence was then measured at E_x_ = 446 nm and E_m_ = 480 nm using a SpectraMax Gemini EM^™^, San Jose, CA, USA [26,62]. Curcumin was used as a positive control, at a concentration of 100 µM.

#### 4.3.3. Antioxidant Activity Assay

The ascorbic derivatives were evaluated for antioxidant activities by DPPH assay at a concentration of 400 µM. The reaction was a mixture of 70 µL of DPPH solution (500 µM in 80% methanol), 10 µL of 80% methanol, and 20 µL of test compound. The reaction was incubated at room temperature in the dark for 30 min. The absorbance was then measured at 517 nm using Infinite 200 Pro^™^ (Tecan) [63]. Ascorbic acid was used as a positive control.

#### 4.3.4. Neuroprotective Activity Assay

##### Cell Culture Preparation

P19 cells were grown in alpha minimal essential medium (α-MEM) supplemented with 7.5% newborn calf serum (NCS), 2.5% fetal bovine serum (FBS), and 1% antibiotics–antimycotic solution in a 5% CO_2_ humidified atmosphere at 37 °C. Cells in monolayer cultures were maintained in exponential growth by subculturing every two days [27].

##### Differentiation of P19 Cells into P19-Derived Neurons

Exponentially grown cultures were trypsinized and dissociated into single cells. P19 cells (2 × 10^6^ cells/mL) were then suspended in 10 mL α-MEM supplemented with 5% FBS, 1% antibiotics–antimycotic solution, and 0.5 µM all transretinoic acid (RA) and seeded onto a 100-mm bacteriological culture dish. The cells formed large aggregates in suspension. After four days of RA treatment, the aggregates were dissociated by 5-mL glass measuring pipette and replated on poly-L-lysine-precoated multiwell plates (the multiwell plates were coated with 50 µg/mL poly-L-lysine, dissolved in PBS overnight, and sterilized under UV light for 30 min) at 7 × 10^4^ cells/mL (150 µL/well in 96-well plate) in α-MEM supplemented with 10% FBS and 1% antibiotics–antimycotic solution and incubated for 24 h. Cytosine-1-β-D-arabinoside or Ara-C (10 µM) was added at day 1 after plating, and the medium was changed every two to three days. The differentiated neuronal cells, P19-derived neurons, were used after day 14 of the differentiation process [27,28,29].

##### Viability Assay

The assay was carried out on P19-derived neurons cultured in a 96-well plate. After 14 days of the differentiation process, the α-MEM, supplemented with 10% FBS, 10 μM Ara-C, and 1% antibiotics–antimycotic solution, was removed and DMSO solutions of the sample, diluted with α-MEM supplemented with 10% FBS and 1% antibiotics–antimycotic solution in the presence of 10 μM Ara-C, were added to produce the concentrations of 10,000, 1000, 100, 10, and 1 nM. A concentration of DMSO was added to the cultures at 0.5% *v/v*. The α-MEM, supplemented with 10% FBS, 10 μM Ara-C, and 1% antibiotics–antimycotic solution, was added to the control wells. Cells were incubated for 18 h at 37 °C. Then, 150 μL of medium was removed, and 50 μL of XTT solution (1 mg/mL XTT in 60 °C α-MEM + 25 μM PMS) was added to the cells. After being incubated at 37 °C for four hours, 100 μL of PBS (phosphate buffer saline solution), pH 7.4, was added. The OD value was determined by using a microplate reader at 450 nm. Data were expressed as mean ± SEM (*n* = 3, each *n* was run in triplicate), with the medium as a control, representing 100% cell viability. The samples that enhanced the survival of cultured neurons more than the control were further investigated for their neuroprotective ability [64].

##### Neuroprotective Assay

The assay was carried out with P19-derived neurons cultured in a 96-well plate by using the serum-deprivation method. DMSO solutions of the samples, diluted with P19SM plus 10 μM Ara-C and α-MEM supplemented with 10 μM Ara-C and 1% antibiotics–antimycotic solution without FBS, were added to give the final concentration of 1 nM, which enhanced survival of cultured neurons more than control. A concentration of DMSO was added to the cultures at 0.5%. P19SM plus 10 μM Ara-C was added into control wells. The α-MEM, supplemented with 10 μM Ara-C and 1% antibiotics–antimycotic solution without FBS, was used to make an oxidative stress condition. The cells were incubated for 18 h at 37 °C. Cell viability was assayed by the XTT reduction method. Data were expressed as mean ± SEM (*n* = 3, each *n* was run in triplicate), with the medium as a control, representing 100% cell viability [30]. Quercetin, at concentration of 1 nM, was used as a positive control [31].

#### 4.3.5. Anti-Inflammatory Activity Assay

Indomethacin, a well-known anti-inflammatory drug, prepared at 50 and 100 mM in DMSO and 0.001% DMSO, was used as the experimental control in both the cell viability assay and the anti-inflammatory gene expression study. The compounds of interest, which are ascorbic acid (**AA**) and ascorbic derivatives (**2c** and **5c**), were prepared as a 10-fold serial dilution in DMSO, from 0.001, 0.01, 0.1, 1, 10 and 100 μM. Prior to testing with cell lines, indomethacin, ascorbic acid, and ascorbic derivatives were mixed with the cell media, DMEM (Dulbecco’s modified Eagle medium), at a ratio of 1:999 µl to produce 50 and 100 µM of indomethacin solutions and 0.001, 0.01, 0.1, 1, 10, and 100 nM of ascorbic acid solutions and ascorbic derivative (**2c** and **5c**) solutions.

##### Cell Culture Preparation

Cell lines, RAW 264.7 (murine) macrophages (ATCC, Manassas, VA, USA), were cultured in DMEM supplemented with 10% fetal bovine serum (FBS), penicillin G (100 U/mL), streptomycin (100 µg/mL), and amphotericin B (0.25 µg/mL). The cell culture was then maintained in an incubator at 37 °C, 5% CO_2_, and 90% relative humidity.

##### Determination of Cell Viability

A 3-(4,5-dimethylthiazol-2-yl)-2,5-diphenyltetrazolium bromide (MTT) reduction assay was used to determine in vitro cell viability. RAW 264.7 starting cells were seeded in 96-well plates at a density of 5 × 10^5^ cells/100 µL per well and then incubated at 37 °C, 5% CO_2_, and 90% relative humidity. Cell lines were monitored to ensure their proper growth and the health of the cells in order to reach 80% confluence within 24 h. DMEM was immediately removed from each well prior to tests with either controls or compounds of interest. To determine cytotoxicity, 100 µL of either 0.001% DMSO, 50 µM indomethacin, 100 µM indomethacin, 0.1% PBS, or the proposed compounds at different concentrations (0.001–100 nM), prepared in DMEM, were separately loaded in each well, and each experiment was carried out in five replicates. After cell lines were preincubated with the testing samples for 24 h, the cell media was replaced with 50 µL MTT solution (1 mg/mL in PBS). The MTT reaction was incubated for four hours; then, 200 µL DMSO was added to each reaction and kept in the dark at room temperature for 10 min. The amount of MTT formazan product formed was determined by measuring absorbance by using a microplate reader at 570 nm. The % cell viability was calculated according to the following equation, with 0.1% PBS as a reference control:

% viability = (O.D. 570 sample/O.D. 570 control) × 100

##### Determination of mRNA Expression Levels of *COX*-2 and *iNOS* Genes

RAW 264.7 starting cells were seeded in six-well plates at a density of 3 × 10^6^ cells/2 mL per well and then incubated at 37 °C, 5% CO_2_, and 90% relative humidity. Cell lines that reached 80% confluence within 48 h were selected for further gene expression study. DMEM was replaced with the test samples, which were prepared as follows: 0.001% DMSO, 50 and 100 μM indomethacin, 10 and 100 nM ascorbic acid, and 10 and 100 nM ascorbic derivatives (**2c** and **5c**). The experiment was conducted in triplicate. After the cell lines were treated with the testing samples for one hour, 2 µg/mL LPS (lipopolysaccharide; Sigma-Aldrich, St. Louis, MO, USA) was added to the cell lines and then maintained in an incubator for another 24 h to induce the cell inflammatory process.

To study the effects of ascorbic acid and ascorbic derivatives (**2c** and **5c**) on the expressions of the inflammatory marker genes, *COX-2* and *iNOS*, total RNA was isolated from the cell lines by a PureLink^™^ RNA Mini Kit (Thermo Fisher Scientific Inc., Waltham, MA, USA). Total RNA quality and quantity were examined by NanoDrop. An equivalent of 2000 ng total RNA from each sample was then reverse-transcribed to produce first-strand complementary DNA (cDNA) using a Tetro cDNA Synthesis Kit (Bioline USA Inc., Memphis, TN, USA), according to the manufacturer’s instruction. Briefly, the total RNA template was mixed with 4 µL 5X RT buffer, 1 µL oligo dT_(18)_, 1 µL dNTP mix (10 µM), 1 µL Rnase inhibitor, and 1 µL reverse transcriptase. Diethylpyrocarbonate (DEPC)-treated water was then added to a mixture to adjust the volume to a final 20-µl reaction. An incubation process to produce first strand cDNA was carried out in a thermal cycler (SensoQuest Labcycler). Primer annealing and strand extending occurred at 45 °C for 30 min and a reverse transcriptase was finally heat-inactivated at 85 °C for 5 min. The corresponding cDNA products were then used as a template in a quantitative polymerase chain reaction (qPCR) to study *COX-2* and *iNOS* gene expressions, with *GAPDH* as a reference gene.

The primer sequences for qPCR quantifications were as follows: *COX-2*: 5′-cccccacagtcaaagacact-3′ (forward), 5′-gagtccatgttccaggagga-3′ (reverse); *iNOS*: 5′-gtcttgcaagctgatggtc-3′ (forward), 5′-catgatggtcacattctgc-3′ (reverse), and *GAPDH*: 5′-caggagcgagaccccactaacat-3′ (forward), 5′-gtcagatccacgacggacacatt-3′ (reverse). These oligonucleotides were synthesized by Invitrogen. qPCR analysis was performed according to the instructions of a SensiFAST^™^ SYBR No-ROX Kit (Bioline USA Inc., USA). Then, 20 µL qPCR reaction was prepared by mixing a 1-µL cDNA template with 0.8 µL forward primer (10 mM), 0.8 µL reverse primer (10 mM), 10 µL SYBR, and 7.4 µL DI water. Gene expression was measured in a 96-well plate using a BIO-RAD iCycler IQ^™^5 detection system. qPCR reaction was initially heated to 95 °C 10 min, and then underwent 40 cycles of 95 °C 15 s and 60 °C 1 min. The modulations of indomethacin, ascorbic acid, and ascorbic derivatives (**2c** and **5c**) to the expression of *COX-2* and *iNOS* were compared against 0.001% DMSO control. Each corresponding expression was computed as a relative fold change using the 2^−delta delta Ct^ method [65], normalized to the *GAPDH* gene.

#### 4.3.6. In Silico Study of Sodium-Dependent Vitamin C Transporter 2 (SVCT2) Binding

The 3D ligands of the synthesized compounds, **2a–2c** and **5a–5c**, from ChemBio3D were geometry optimized using the Guassian 09w program with the B3LYP/6-31G(d,p) method. Subsequently, the aromatic bonds and rotatable bonds of each molecule were identified. All hydrogen atoms and Gasteiger’s charges were assigned through the usage of AutoDockTools-1.5.6. These conformations were then saved in pdbqt file format.

The crystallographic model of the bacterial vitamin c transporter (pdb code: 4RP9) [24] was used in the present study since structural information of human SVCTs is not available. All cocrystallized ligands, metal ions, and water molecules were removed from the structure through Discovery Studio 2017R2. All hydrogen atoms and Gasteiger’s charges were assigned using AutoDockTool-1.5.6. Then, this pretreated protein was saved in pdb file format. A grid box was generated from AutoGrid 4.0, presenting AutoDockTool-1.5.6 as a graphical interface, which is defined as a coordinate grid center. The size of the grid box was set as 60 × 60 × 60 Å^3^, with grid spacing of 0.375 Å. All docking simulations were performed utilizing the AutoDock 4.0 program. Total binding energies of 100 docked conformers, generated from 300 population sizes, were calculated based on Lamarkian genetic algorithms (LGA).

## Figures and Tables

**Table 1:** Multifunctional in vitro activities of ascorbic derivatives at a concentration of 400 µM.

Compound	BACE1		Amyloid Aggregation		Antioxidant	
	%Inhibition (±SEM)	IC_50_ (μM)	%Inhibition (±SEM)	IC_50_ (μM)	%Inhibition (±SEM)	IC_50_ (μM)
**2a**	6.80 ± 2.69	-	53.01 ± 0.60	318.60	66.54 ± 0.56 *	206.50
**2b**	6.29 ± 0.98	-	50.99 ± 2.12 *	318.80	62.81 ± 2.62 *	155.70
**2c**	23.49 ± 1.66 *	725.70	71.79 ± 1.42	92.33	94.67 ± 0.50	72.26
**5a**	ND	-	34.53 ± 3.62 *	-	78.42 ± 0.93 *	91.15
**5b**	ND	-	19.58 ± 1.83 *	-	77.37 ± 1.60 *	103.10
**5c**	27.33 ± 1.41 *	593.10	68.26 ± 0.67	136.00	85.84 ± 2.43	62.89
Ascorbic acid	7.84 ± 1.75	-	63.44 ± 0.87	146.10	94.85 ± 0.62	21.28
BACE1 inhibitor I (100 μM)	99.14 ± 0.11 *	-	-	-	-	-
Curcumin (100 μM)	-	-	94.45 ± 1.15	-	-	-

ND indicates not detected. All data are presented as means ± SEM (*n* = 3). Asterisk denotes a significant difference compared with the ascorbic acid of each group, based on paired *t*-test statistical analysis by using Microsoft Excel (* *p* ≤ 0.01).

## Data Availability

Data sharing not applicable.

## Supplementary Materials

Table S1: The percent yields and structures of synthesized compounds, Figures S1–S12: NMR spectrums (300 MHz) of synthesized compounds, Figure S13: Viability assay by XTT of ascorbic derivatives at concentration of 1 to 10^4^ nM; Figure S14: Effects of ascorbic acid (A), **2c** (B), and **5c** (C) on the viability of RAW 264.7.

## Sample Avalability

The sample of compounds are not available.

## References

[1] Dementia Statistics. Alzheimer’s Disease International. https://www.alzint.org/about/dementia-facts-figures/dementia-statistics.

[2] Mattson M.P. (2004). Pathways towards and away from Alzheimer’s disease. Nature.

[3] Yatin S.M., Yatin M., Aulick T., Ain K.B., Butterfield D.A. (1999). Alzheimer’s amyloid beta-peptide associated free radicals increase rat embryonic neuronal polyamine uptake and ornithine decarboxylase activity: Protective effect of vitamin E. Neurosci. Lett..

[4] Heneka M.T., Golenbock D.T., Latz E. (2015). Innate immunity in Alzheimer’s disease. Nat. Immunol..

[5] Chen G.-F., Xu T.-H., Yan Y., Zhou Y.-R., Jiang Y., Melcher K., Xu H.E. (2017). Amyloid beta: Structure, biology and structure-based therapeutic development. Acta Pharmacol. Sin..

[6] Pasinetti G.M., Aisen P.S. (1998). Cyclooxygenase-2 expression is increased in frontal cortex of Alzheimer’s disease brain. Neuroscience.

[7] Nathan C., Calingasan N., Nezezon J., Ding A., Lucia M.S., La Perle K., Fuortes M., Lin M., Ehrt S., Kwon N.S. (2005). Protection from Alzheimer’s-like disease in the mouse by genetic ablation of inducible nitric oxide synthase. J. Exp. Med..

[8] Van Marum R.J. (2008). Current and future therapy in Alzheimer’s disease. Fundam. Clin. Pharmacol..

[9] Gabr M.T., Ibrahim M.M. (2019). Multitarget therapeutic strategies for Alzheimer’s disease. Neural Regen. Res..

[10] Harrison F.E., May J.M. (2009). Vitamin C function in the brain: Vital role of the ascorbate transporter SVCT2. Free Radic. Biol. Med..

[11] Kook S.-Y., Lee K.-M., Kim Y., Cha M.-Y., Kang S., Baik S.H., Lee H., Park R., Mook-Jung I. (2014). High-dose of vitamin C supplementation reduces amyloid plaque burden and ameliorates pathological changes in the brain of 5XFAD mice. Cell Death Dis..

[12] Lykkesfeldt J., Tveden-Nyborg P. (2019). The Pharmacokinetics of Vitamin C. Nutrients.

[13] Kübler W., Gehler J. (1970). Kinetics of intestinal absorption of ascorbic acid. Calculation of non-dosage-dependent absorption processes. Int. Z. Vitam..

[14] Mayersohn M. (1972). Ascorbic acid absorption in man—Pharmacokinetic implications. Eur. J. Pharmacol..

[15] Hasselholt S., Tveden-Nyborg P., Lykkesfeldt J. (2015). Distribution of vitamin C is tissue specific with early saturation of the brain and adrenal glands following differential oral dose regimens in guinea pigs. Br. J. Nutr..

[16] Levine M., Rumsey S.C., Daruwala R., Park J.B., Wang Y. (1999). Criteria and Recommendations for Vitamin C Intake. JAMA.

[17] Levine M., Padayatty S.J., Espey M.G. (2011). Vitamin C: A Concentration-Function Approach Yields Pharmacology and Therapeutic Discoveries. Adv. Nutr..

[18] Chen Q., Espey M.G., Sun A.Y., Lee J.-H., Krishna M.C., Shacter E., Choyke P.L., Pooput C., Kirk K.L., Buettner G.R. (2007). Ascorbate in pharmacologic concentrations selectively generates ascorbate radical and hydrogen peroxide in extracellular fluid in vivo. Proc. Natl. Acad. Sci. USA.

[19] Macan A.M., Kraljević T.G., Raić-Malić S. (2019). Therapeutic Perspective of Vitamin C and Its Derivatives. Antioxidants.

[20] Timoshnikov V.A., Kobzeva T.V., Polyakov N.E., Kontoghiorghes G.J. (2020). Redox Interactions of Vitamin C and Iron: Inhibition of the Pro-Oxidant Activity by Deferiprone. Int. J. Mol. Sci..

[21] Teponnou G.A.K., Joubert J., Malan S.F. (2017). Tacrine, Trolox and Tryptoline as Lead Compounds for the Design and Synthesis of Multi-target Agents for Alzheimer’s Disease Therapy. Open Med. Chem. J..

[22] Herraiz T., González D., Ancín-Azpilicueta C., Arán V.J., Guillén H. (2010). β-Carboline alkaloids in Peganum harmala and inhibition of human monoamine oxidase (MAO). Food Chem. Toxicol..

[23] Frost D., Meechoovet B., Wang T., Gately S., Giorgetti M., Shcherbakova I., Dunckley T. (2011). β-carboline compounds, including harmine, inhibit DYRK1A and tau phosphorylation at multiple Alzheimer’s disease-related sites. PLoS ONE.

[24] Zheng W., Wang S.Y. (2001). Antioxidant Activity and Phenolic Compounds in Selected Herbs. J. Agric. Food Chem..

[25] Reinke A.A., Gestwicki J.E. (2007). Structure-Activity Relationships of Amyloid Beta-Aggregation Inhibitors Based on Curcumin: Influence of Linker Length and Flexibility. Chem. Biol. Drug Des..

[26] Jiaranaikulwanitch J., Govitrapong P., Fokin V.V., Vajragupta O. (2012). From BACE1 Inhibitor to Multifunctionality of Tryptoline and Tryptamine Triazole Derivatives for Alzheimer’s Disease. Molecules.

[27] Jones-Villeneuve E.M.V., McBurney M.W., Rogers K.A., Kalnins V.I. (1982). Retinoic acid induces embryonal carcinoma cells to differentiate into neurons and glial cells. J. Cell Biol..

[28] Jones-Villeneuve E.M., Rudnicki M.A., Harris J.F., McBurney M.W. (1983). Retinoic acid-induced neural differentiation of embryonal carcinoma cells. Mol. Cell. Biol..

[29] MacPherson P.A., McBurney M.W. (1995). P19 Embryonal Carcinoma Cells: A Source of Cultured Neurons Amenable to Genetic Manipulation. Methods.

[30] Tadtong S., Meksuriyen D., Tanasupawat S., Isobe M., Suwanborirux K. (2007). Geldanamycin derivatives and neuroprotective effect on cultured P19-derived neurons. Bioorg. Med. Chem. Lett..

[31] Tangsaengvit N., Kitphati W., Tadtong S., Bunyapraphatsara N., Nukoolkarn V. (2013). Neurite Outgrowth and Neuroprotective Effects of Quercetin from Caesalpinia mimosoides Lamk. on Cultured P19-Derived Neurons. Evid. Based Complement. Alternat. Med..

[32] Tadtong S., Kanlayavattanakul M., Lourith N. (2013). Neuritogenic and Neuroprotective Activities of Fruit Residues. Nat. Prod. Commun..

[33] Panyatip P., Tadtong S., Sousa E., Puthongking P. (2020). BACE1 Inhibitor, Neuroprotective, and Neuritogenic Activities of Melatonin Derivatives. Sci. Pharm..

[34] Su X., Shen Z., Yang Q., Sui F., Pu J., Ma J., Ma S., Yao D., Ji M., Hou P. (2019). Vitamin C kills thyroid cancer cells through ROS-dependent inhibition of MAPK/ERK and PI3K/AKT pathways via distinct mechanisms. Theranostics.

[35] Clément M.-V., Ramalingam J., Long L.H., Halliwell B. (2001). The In Vitro Cytotoxicity of Ascorbate Depends on the Culture Medium Used to Perform the Assay and Involves Hydrogen Peroxide. Antioxid. Redox Signal..

[36] Liu P.-Y., Jiang N., Zhang J., Wei X., Lin H.-H., Yu X.-Q. (2006). The Oxidative Damage of Plasmid DNA by Ascorbic Acid Derivatives In Vitro: The First Research on the Relationship between the Structure of Ascorbic Acid and the Oxidative Damage of Plasmid DNA. Chem. Biodivers..

[37] Liu Y., Liu C., Li J. (2020). Comparison of Vitamin C and Its Derivative Antioxidant Activity: Evaluated by Using Density Functional Theory. ACS Omega.

[38] Cesarini E., Cerioni L., Canonico B., Di Sario G., Guidarelli A., Lattanzi D., Savelli D., Guescini M., Nasoni M.G., Bigini N. (2018). Melatonin protects hippocampal HT22 cells from the effects of serum deprivation specifically targeting mitochondria. PLoS ONE.

[39] Lee J.-S., Lee M.-O., Moon B.-H., Shim S.H., Fornace A.J., Cha H.-J. (2009). Senescent Growth Arrest in Mesenchymal Stem Cells Is Bypassed by Wip1-Mediated Downregulation of Intrinsic Stress Signaling Pathways. Stem Cells.

[40] Biteghe F.N., Davids L.M. (2017). A combination of photodynamic therapy and chemotherapy displays a differential cytotoxic effect on human metastatic melanoma cells. J. Photochem. Photobiol. B Biol..

[41] Sun M., Wang M., Chen M., Dagnaes-Hansen F., Le D.Q.S., Baatrup A., Horsman M.R., Kjems J., Bünger C.E. (2015). A tissue-engineered therapeutic device inhibits tumor growth in vitro and in vivo. Acta Biomater..

[42] Bose S., Sarkar N., Vahabzadeh S. (2019). Sustained release of vitamin C from PCL coated TCP induces proliferation and differentiation of osteoblast cells and suppresses osteosarcoma cell growth. Mater. Sci. Eng. C Mater. Biol. Appl..

[43] Sommer F., Kobuch K., Brandl F., Wild B., Framme C., Weiser B., Tessmar J., Gabel V.-P., Blunk T., Goepferich A. (2007). Ascorbic Acid Modulates Proliferation and Extracellular Matrix Accumulation of Hyalocytes. Tissue Eng..

[44] Garciá-Martínez O., De Luna-Bertos E., Ramos-Torrecillas J., Ruiz C., Milia E., Lorenzo M.L., Jiménez B., Sánchez-Ortiz A., Rivas A. (2016). Phenolic Compounds in Extra Virgin Olive Oil Stimulate Human Osteoblastic Cell Proliferation. PLoS ONE.

[45] Zadeh K.M., Luyt A.S., Zarif L., Augustine R., Hasan A., Messori M., Hassan M.K., Yalcin H.C. (2019). Electrospun polylactic acid/date palm polyphenol extract nanofibres for tissue engineering applications. Emergent Mater..

[46] Fiorillo M., Tóth F., Sotgia F., Lisanti M.P. (2019). Doxycycline, Azithromycin and Vitamin C (DAV): A potent combination therapy for targeting mitochondria and eradicating cancer stem cells (CSCs). Aging.

[47] Jiaranaikulwanitch J., Tadtong S., Govitrapong P., Fokin V.V., Vajragupta O. (2017). Neuritogenic activity of bi-functional bis-tryptoline triazole. Bioorg. Med. Chem..

[48] Trigo D., Goncalves M.B., Corcoran J.P.T. (2019). The regulation of mitochondrial dynamics in neurite outgrowth by retinoic acid receptor β signaling. FASEB J..

[49] Abdollahzad H., Eghtesadi S., Nourmohammadi I., Khadem-Ansari M., Nejad-Gashti H., Esmaillzadeh A. (2009). Effect of Vitamin C Supplementation on Oxidative Stress and Lipid Profiles in Hemodialysis Patients. Int. J. Vitam. Nutr. Res..

[50] Yimcharoen M., Kittikunnathum S., Suknikorn C., Nak-On W., Yeethong P., Anthony T.G., Bunpo P. (2019). Effects of ascorbic acid supplementation on oxidative stress markers in healthy women following a single bout of exercise. J. Int. Soc. Sports Nutr..

[51] Kawashima A., Sekizawa A., Koide K., Hasegawa J., Satoh K., Arakaki T., Takenaka S., Matsuoka R. (2015). Vitamin C Induces the Reduction of Oxidative Stress and Paradoxically Stimulates the Apoptotic Gene Expression in Extravillous Trophoblasts Derived from First-Trimester Tissue. Reprod. Sci..

[52] Ho L., Purohit D., Haroutunian V., Luterman J.D., Willis F., Naslund J., Buxbaum J.D., Mohs R.C., Aisen P.S., Pasinetti G.M. (2001). Neuronal Cyclooxygenase 2 Expression in the Hippocampal Formation as a Function of the Clinical Progression of Alzheimer Disease. Arch. Neurol..

[53] Han S.S., Kim K., Hahm E.R., Lee S.J., Surh Y.J., Park H.K., Kim W.S., Jung C.W., Lee M.H., Park K. (2004). L-ascorbic acid represses constitutive activation of NF-κB and COX-2 expression in human acute myeloid leukemia, HL-60. J. Cell. Biochem..

[54] Wu F., Wilson J.X., Tyml K. (2003). Ascorbate inhibits iNOS expression and preserves vasoconstrictor responsiveness in skeletal muscle of septic mice. Am. J. Physiol. Regul. Integr. Comp. Physiol..

[55] Caprile T., Salazar K., Astuya A., Cisternas P., Silva-Alvarez C., Montecinos H., Millán C., García M.D.L.A., Nualart F. (2009). The Na+-dependent l-ascorbic acid transporter SVCT2 expressed in brainstem cells, neurons, and neuroblastoma cells is inhibited by flavonoids. J. Neurochem..

[56] Manfredini S., Pavan B., Vertuani S., Scaglianti M., Compagnone D., Biondi C., Scatturin A., Tanganelli S., Ferraro L., Prasad P. (2002). Design, synthesis and activity of ascorbic acid prodrugs of nipecotic, kynurenic and diclophenamic acids, liable to increase neurotropic activity. J. Med. Chem..

[57] Yue Q., Peng Y., Zhao Y., Lu R., Fu Q., Chen Y., Yang Y., Hai L., Guo L., Wu Y. (2018). Dual-targeting for brain-specific drug delivery: Synthesis and biological evaluation. Drug Deliv..

[58] Luo P., Yu X., Wang W., Fan S., Li X., Wang J. (2015). Crystal structure of a phosphorylation-coupled vitamin C transporter. Nat. Struct. Mol. Biol..

[59] Olabisi A.O., Wimalasena K. (2004). Rational Approach to Selective and Direct 2-O-Alkylation of 5,6-O-Isopropylidine-l-ascorbic Acid. J. Org. Chem..

[60] Jiaranaikulwanitch J., Boonyarat C., Fokin V.V., Vajragupta O. (2010). Triazolyl tryptoline derivatives as β-secretase inhibitors. Bioorg. Med. Chem. Lett..

[61] Ermolieff J., Loy J.A., Koelsch G., Tang J. (2000). Proteolytic Activation of Recombinant Pro-Memapsin 2 (Pro-β-Secretase) Studied with New Fluorogenic Substrates. Biochemistry.

[62] LeVine H. (1993). Thioflavine T interaction with synthetic Alzheimer’s disease beta-amyloid peptides: Detection of amyloid aggregation in solution. Protein Sci..

[63] Sharma O.P., Bhat T.K. (2009). DPPH antioxidant assay revisited. Food Chem..

[64] Iacovitti L., Stull N.D., Johnston K. (1997). Melatonin rescues dopamine neurons from cell death in tissue culture models of oxidative stress. Brain Res..

[65] Livak K.J., Schmittgen T.D. (2001). Analysis of Relative Gene Expression Data Using Real-Time Quantitative PCR and the 2−ΔΔCT Method. Methods.

