# Supplementary material for: Novel Multifunctional Ascorbic Triazole Derivatives for Amyloidogenic Pathway Inhibition, Anti-Inflammation, and Neuroprotection
Source: Molecules. 2021 Mar 12;26(6):1562. doi: 10.3390/molecules26061562 (PMC7999550; doi:10.3390/molecules26061562)
Supplement: Supplementary file 1 [file molecules-26-01562-s001.pdf]

Novel Multifunctional Ascorbic Triazole Derivatives for Amyloidogenic Pathway Inhibition, Anti-inflammation, and Neuroprotection

1. Synthesis

The percent yields of synthesized compounds were report in **table S1**. The ^{1}H-NMR and ^{1}C-NMR spectrums of final synthesized compounds were shown in figures **S1-S12**.

Table S1. The percent yields and structures of synthesized compounds.

Compound	Structure	% yield
**2**	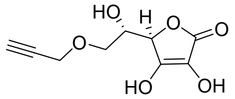	29.00
**3**	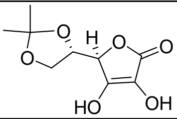	65.88
**4**	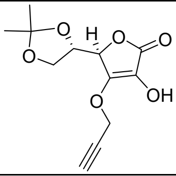	51.13
**5**	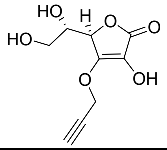	86.14
**a**	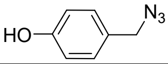	16.15
**b**	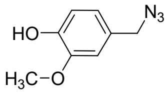	13.51
**In-c1**	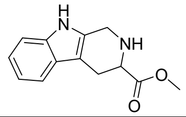	99.64
**In-c2**	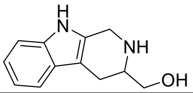	58.32
**In-c4**	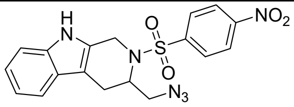	78.28
**c**	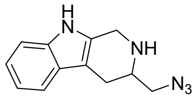	41.19
**2a**	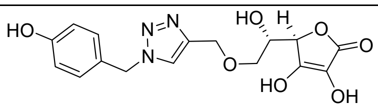	8.58
**2b**	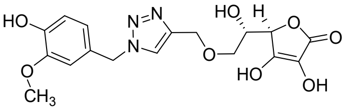	12.30
**2c**	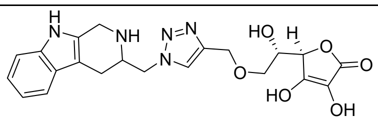	24.52

Table S1. The percent yields and structures of synthesized compounds (cont.).

Compound	Structure	% yield
**5a**	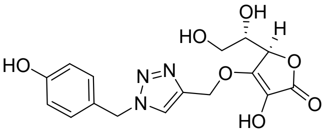	13.70
**5b**	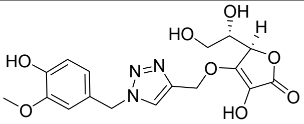	24.61
**5c**	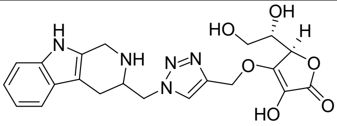	13.32

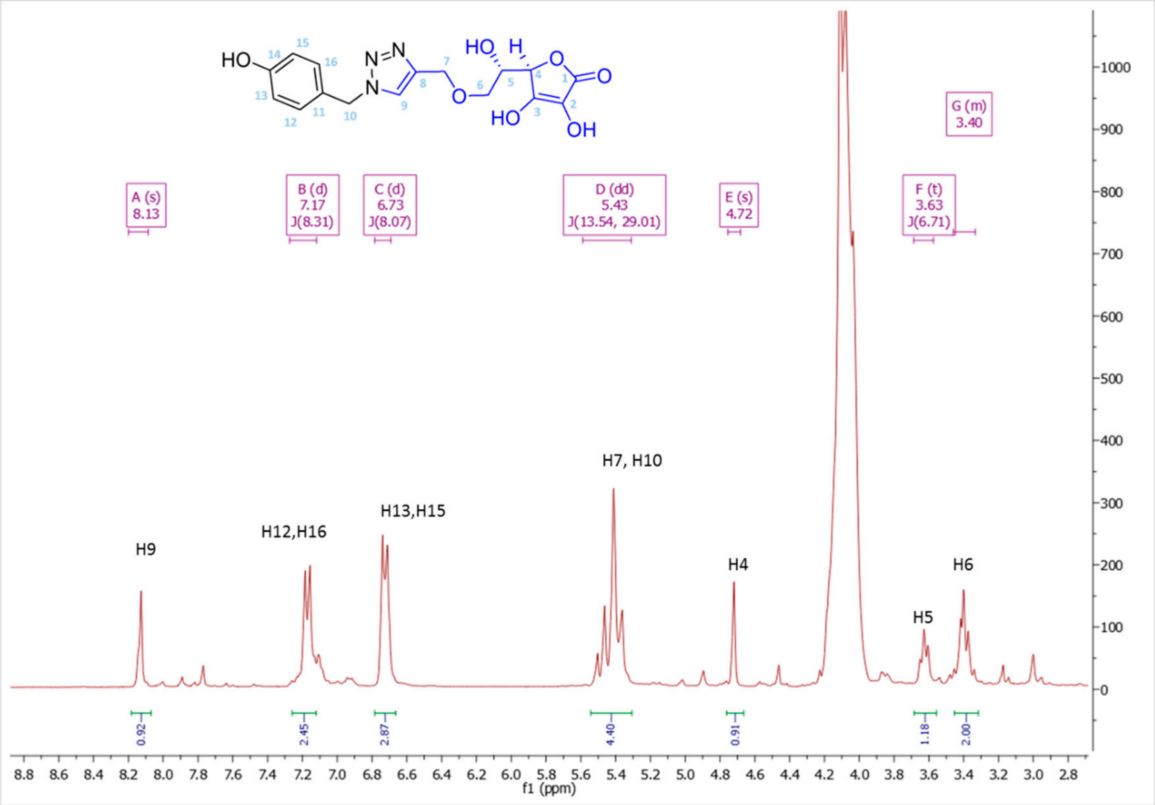

Figure S1. ^{1}HNMR spectrum (300 MHz) of compound **[2a]** in DMSO-d6.

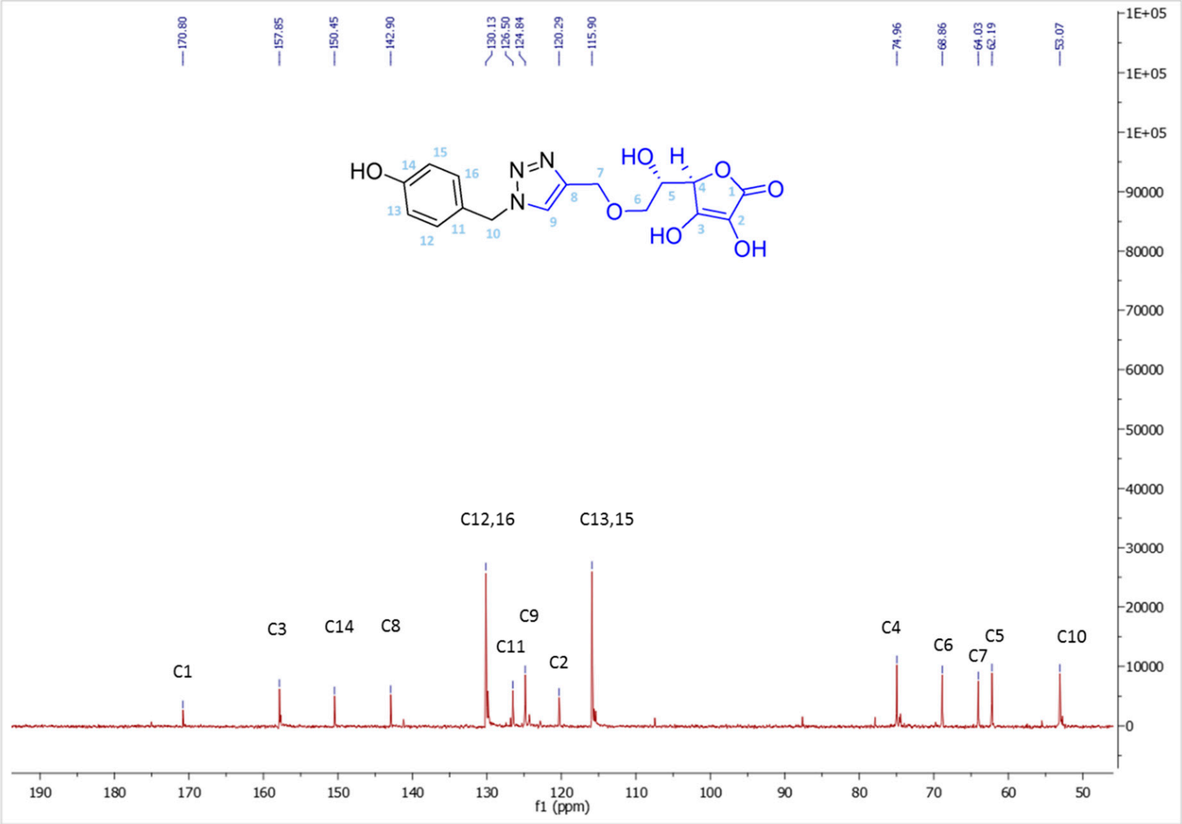

Figure S2. ^{13}CNMR spectrum (300 MHz) of compound **[2a]** in DMSO-d$_6$.

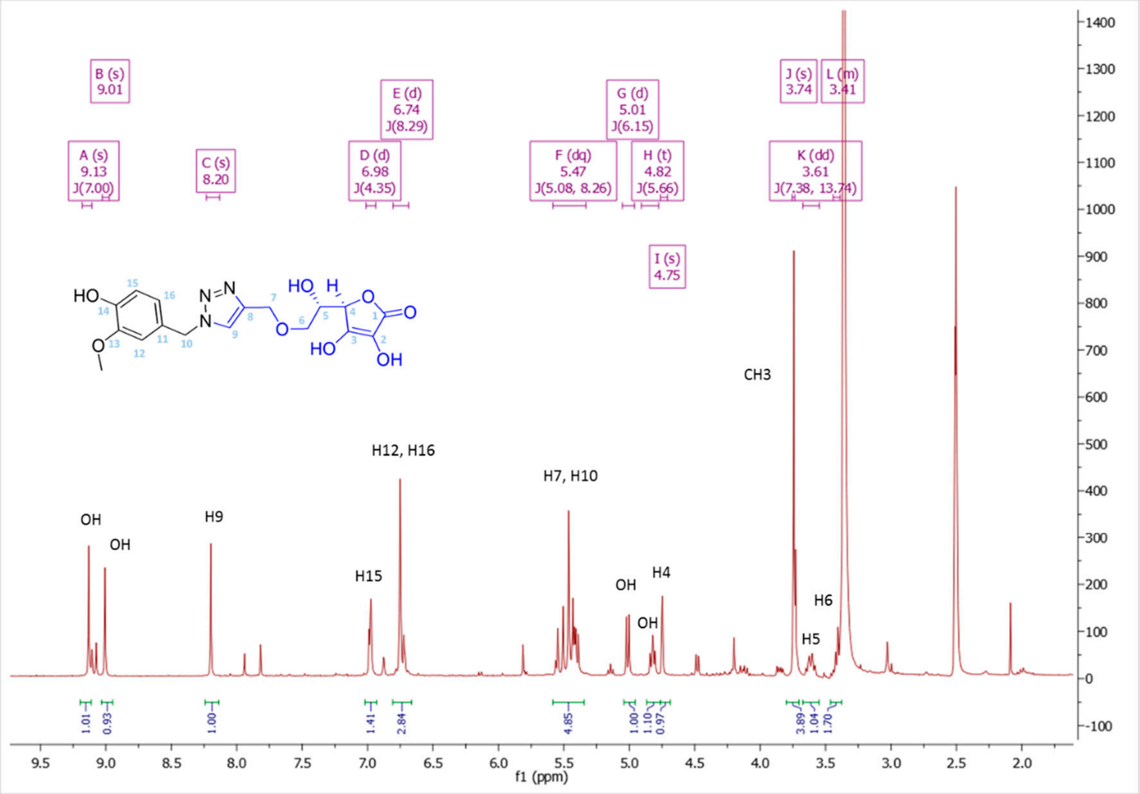

Figure S3. ^{1}HNMR spectrum (300 MHz) of compound **[2b]** in DMSO-d$_6$.

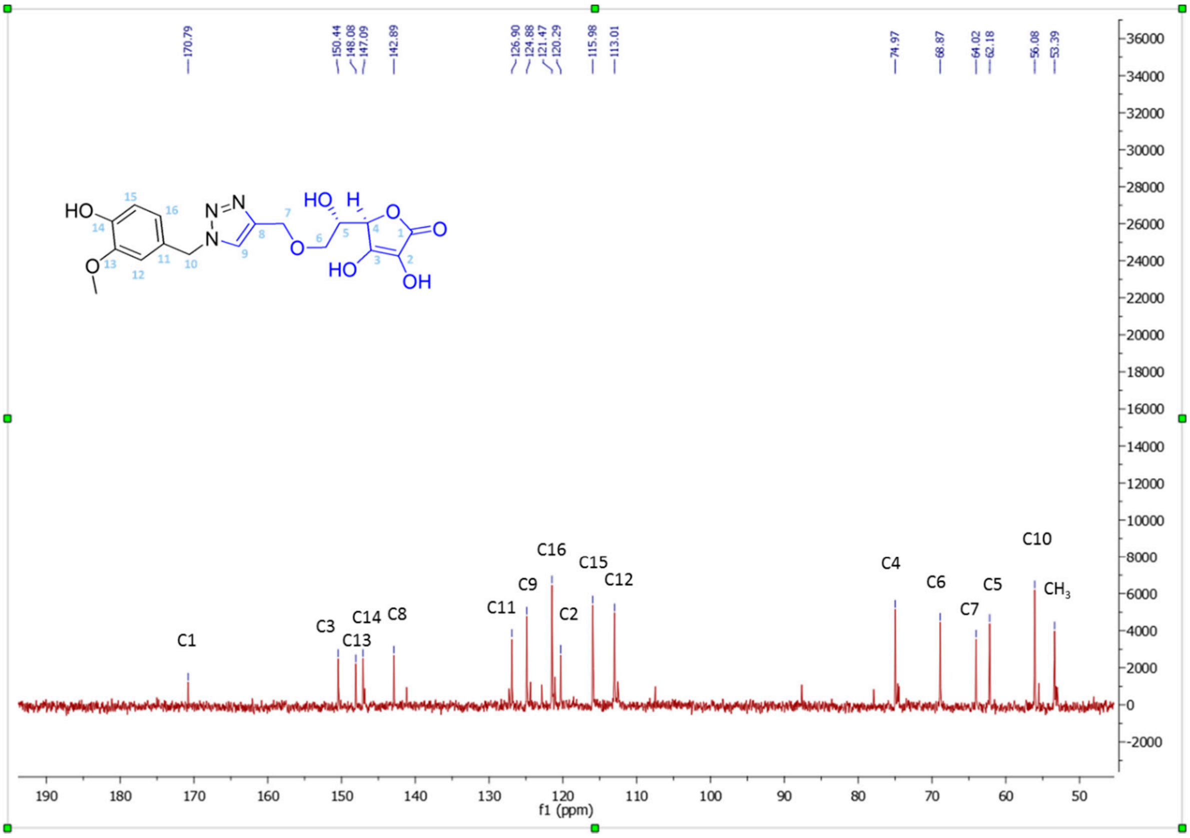

Figure S4. ^{13}CNMR spectrum (300 MHz) of compound **[2b]** in DMSO-d$_6$.

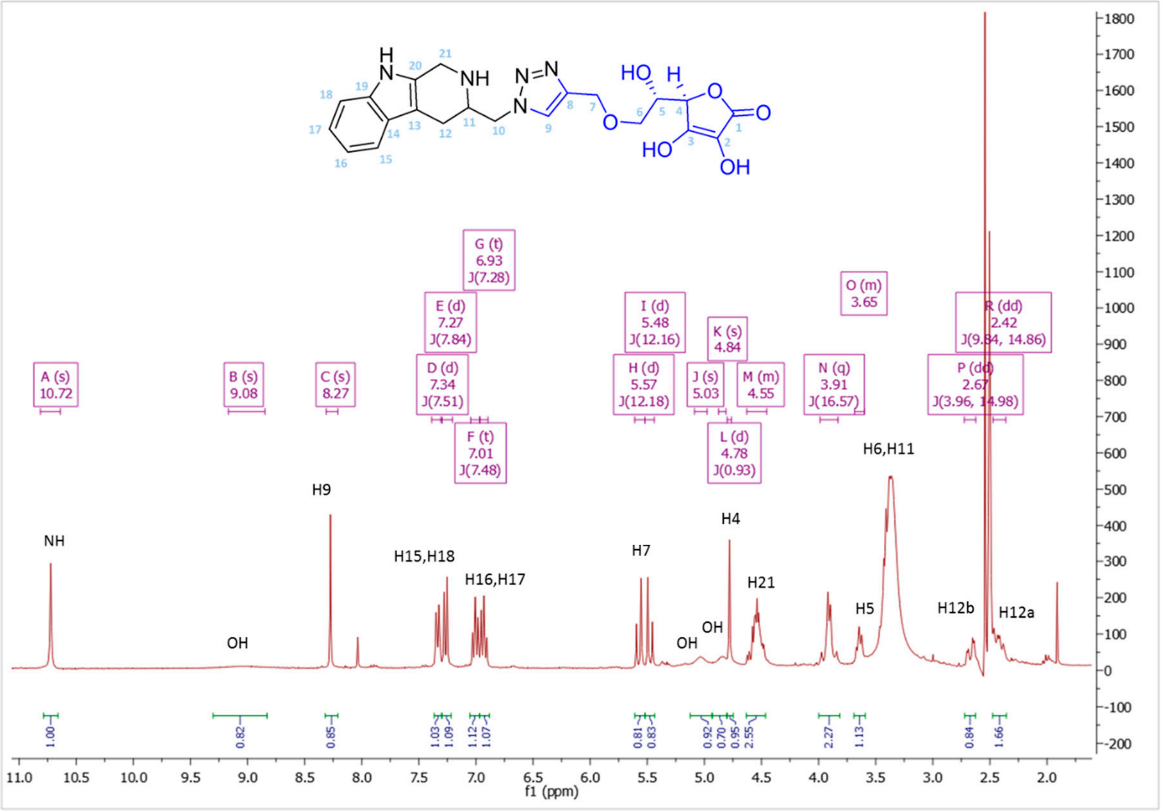

Figure S5. ^{1}HNMR spectrum (300 MHz) of compound **[2c]** in DMSO-d$_6$.

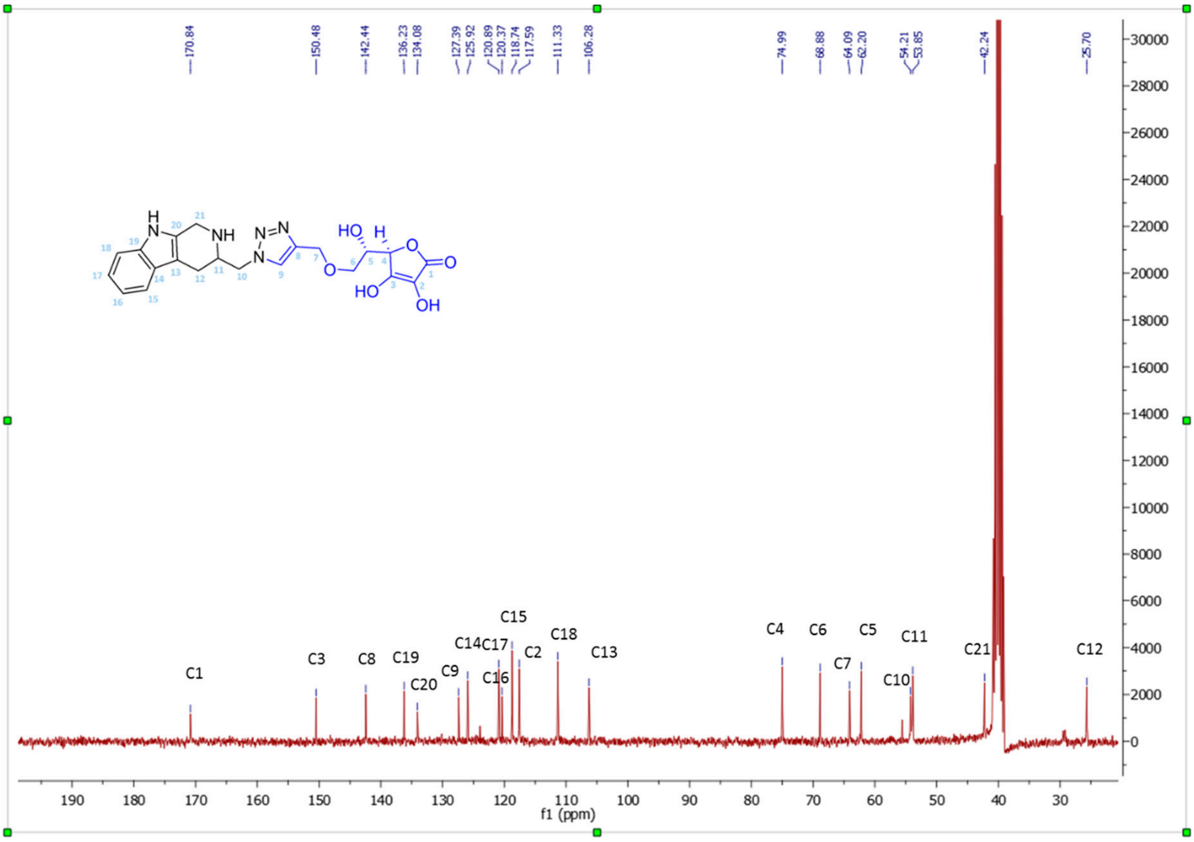

Figure S6. ^{13}CNMR spectrum (300 MHz) of compound **[2c]** in DMSO-d_6.

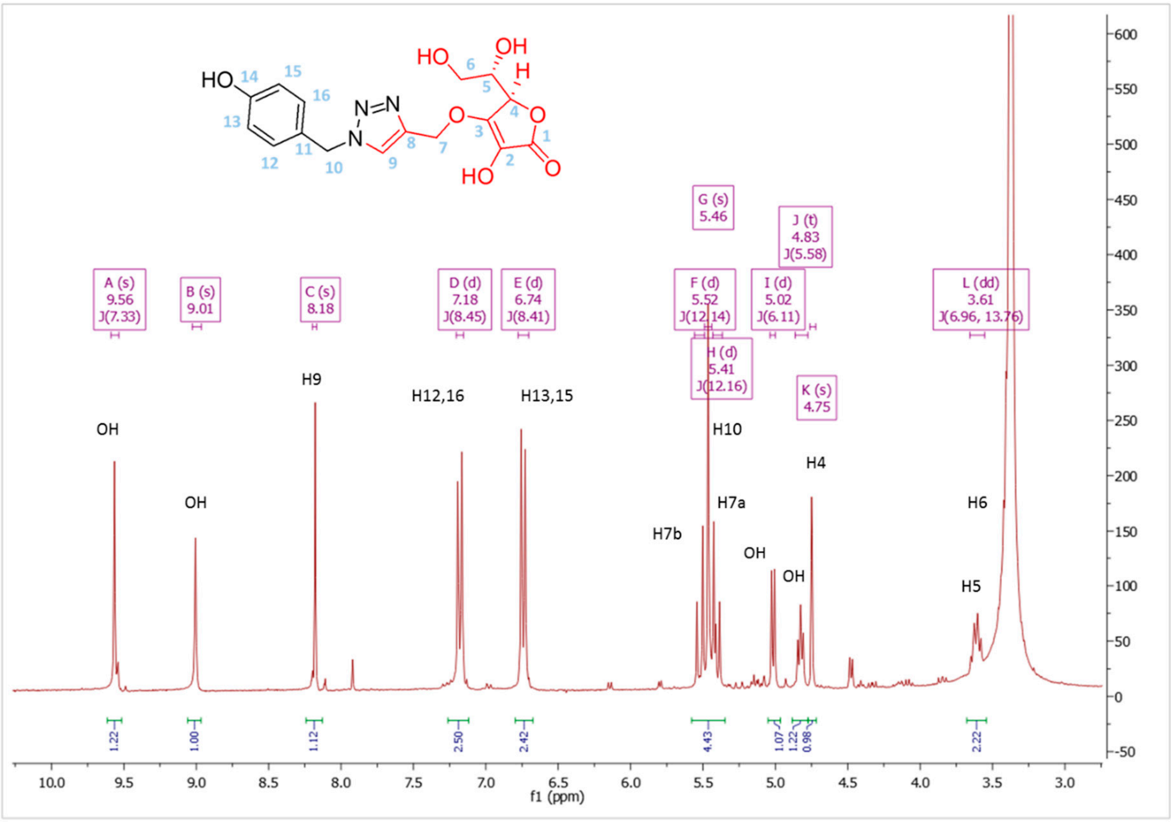

Figure S7. ^{1}HNMR spectrum (300 MHz) of compound **[5a]** in DMSO-d6.

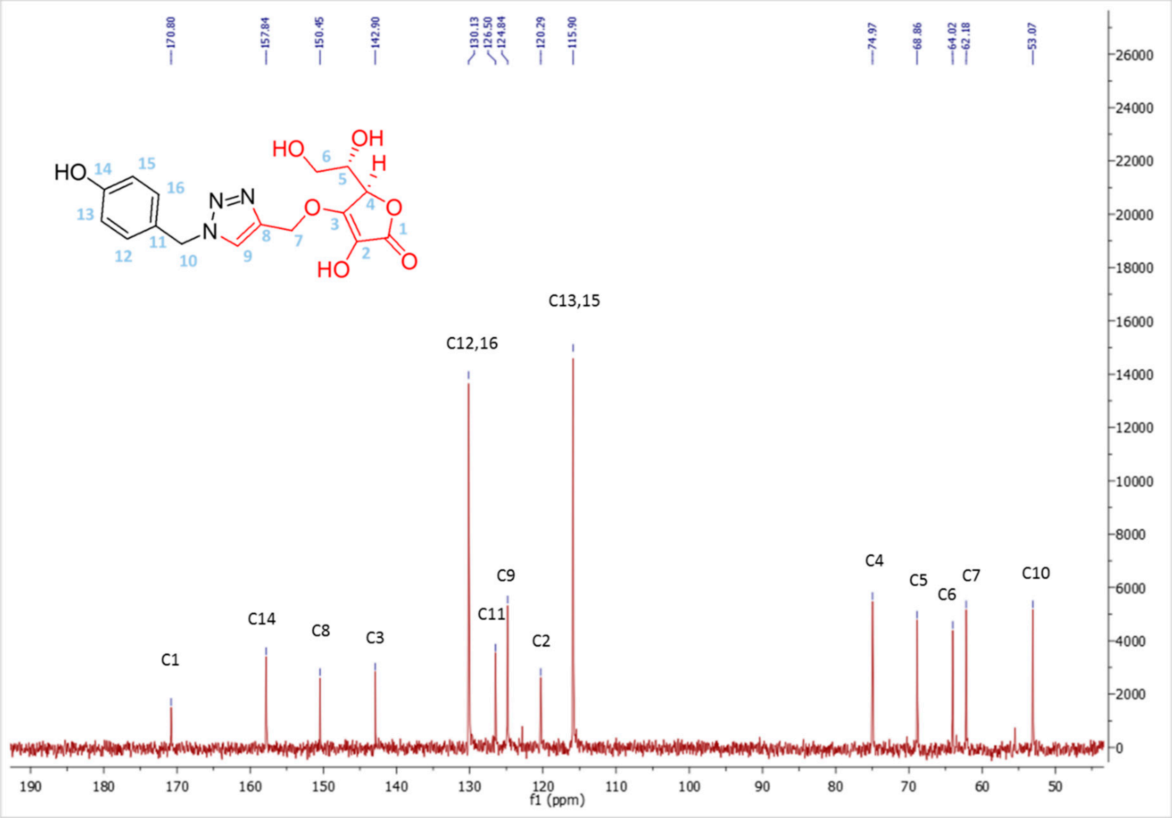

Figure S8. ^{13}CNMR spectrum (300 MHz) of compound **[5a]** in DMSO-d6.

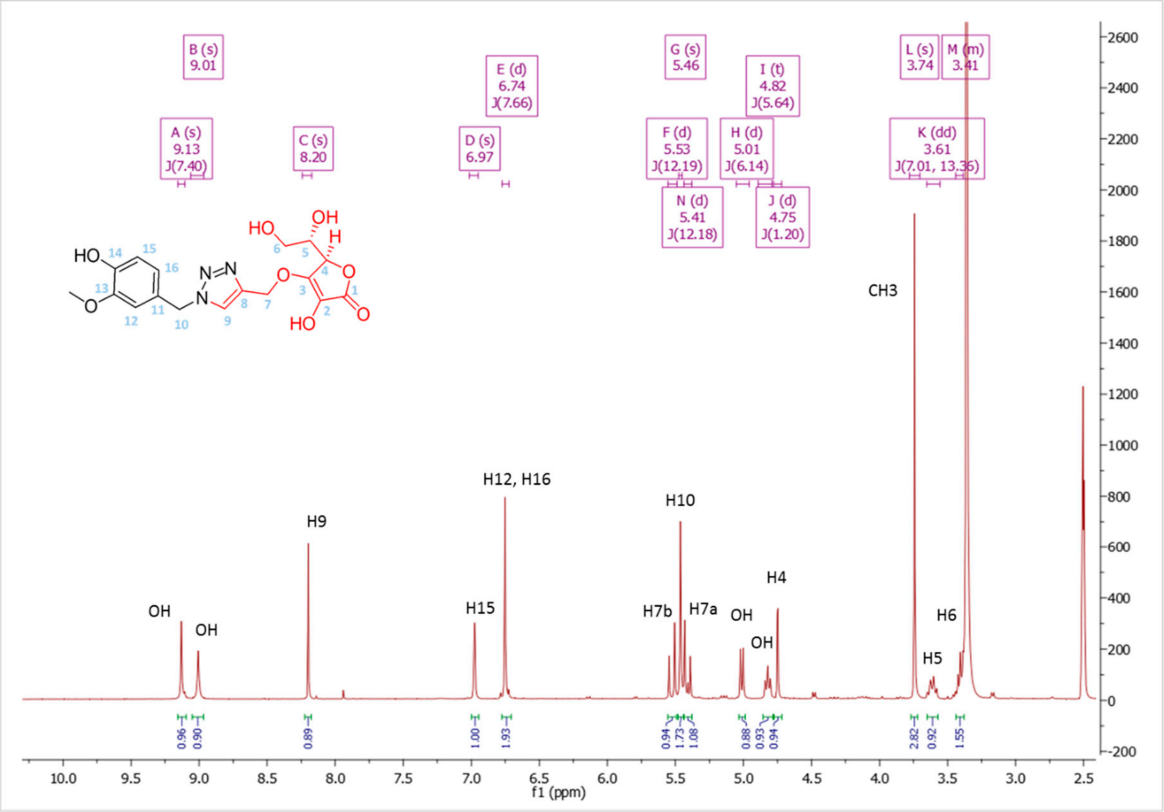

Figure S9. ^{1}HNMR spectrum (300 MHz) of compound **[5b]** in DMSO-d6.

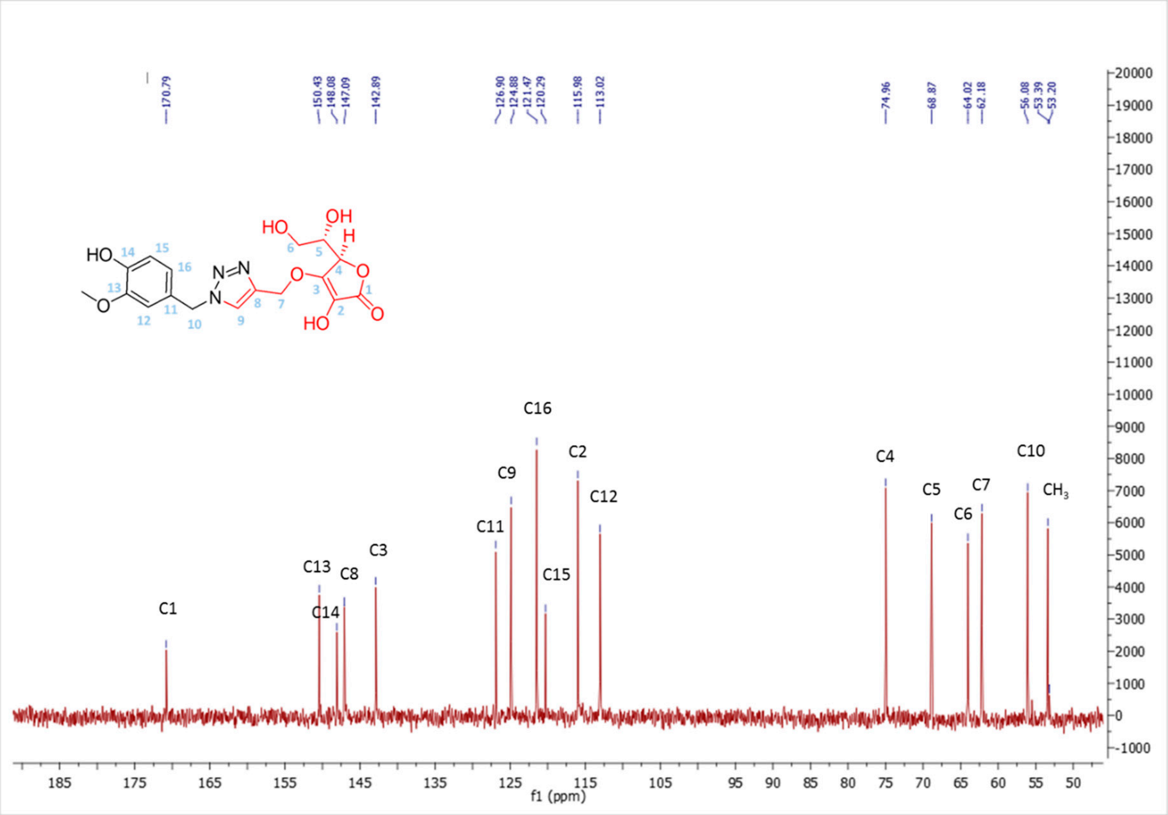

Figure S10. ^{13}CNMR spectrum (300 MHz) of compound **[5b]** in DMSO-d$_6$.

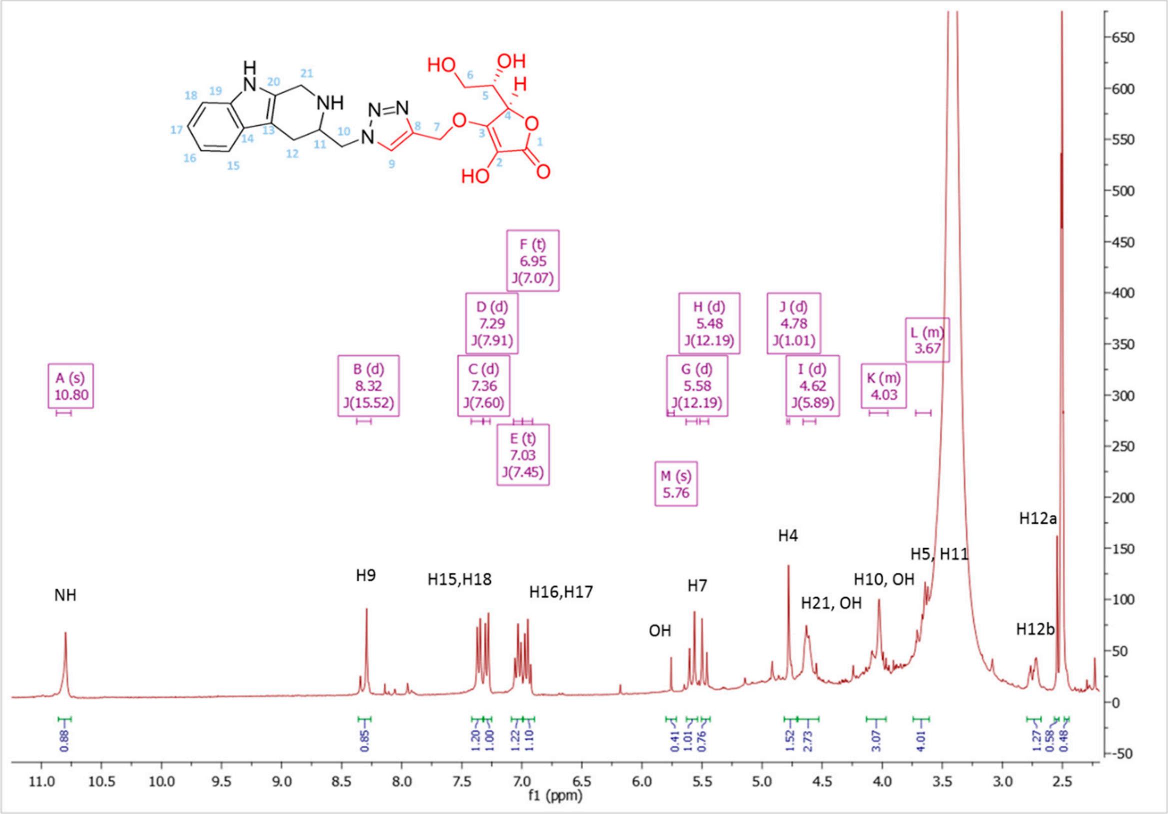

Figure S11. ^{1}HNMR spectrum (300 MHz) of compound **[5c]** in DMSO-d$_6$.

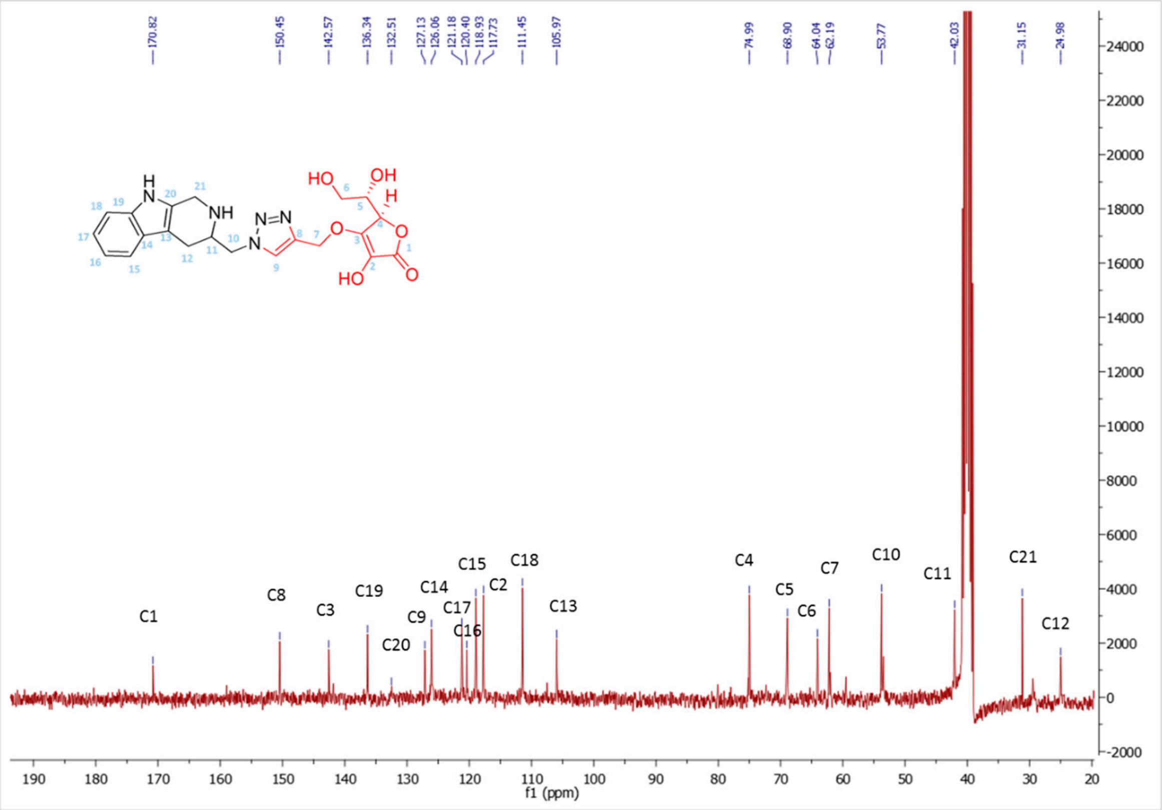

Figure S12. ^{13}CNMR spectrum (300 MHz) of compound **[5c]** in DMSO-d_6.

2. Biological activity assays

Cell viability assays for neurotoxicity assessments of ascorbic acid and derivatives (**2a-2c** and **5a-5c**) were conducted at various concentrations of 1 to 10^4 nM on P19-derived neurons and measured by XTT method. All compounds except ascorbic acid had cell viabilities higher than 100% at 1 nM (Figure S13).

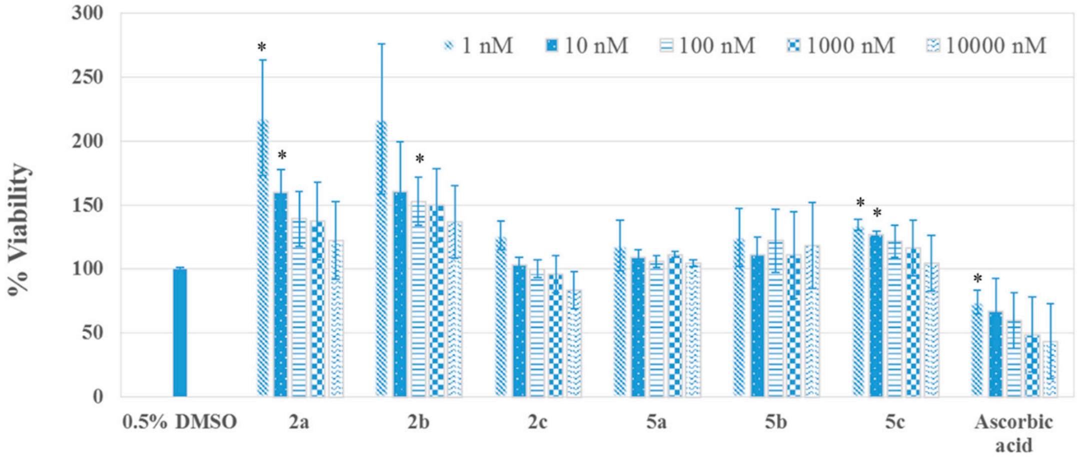

Figure S13. Effects of ascorbic acid and derivatives (**2a-2c** and **5a-5c**) at concentrations of 1 to 10^4 nM on the viability of P19-derived neurons. The cytotoxicity was evaluated by XTT assay. Each bar is presented as mean ± SD (n = 3). * indicates significant differences with 0.5% DMSO (p-value < 0.05) based on t-test by using Microsoft Excel version 2013.

Cell viability assays for anti-inflammatory assessments of ascorbic acid, **2c**, and **5c** were performed at various concentrations of 0.001 to 100 nM on RAW 264.7 and measured by MTT method. The ascorbic acid, **2c**, and **5c** did not exhibit cytotoxicity after 24-hour exposure (Figure S14).

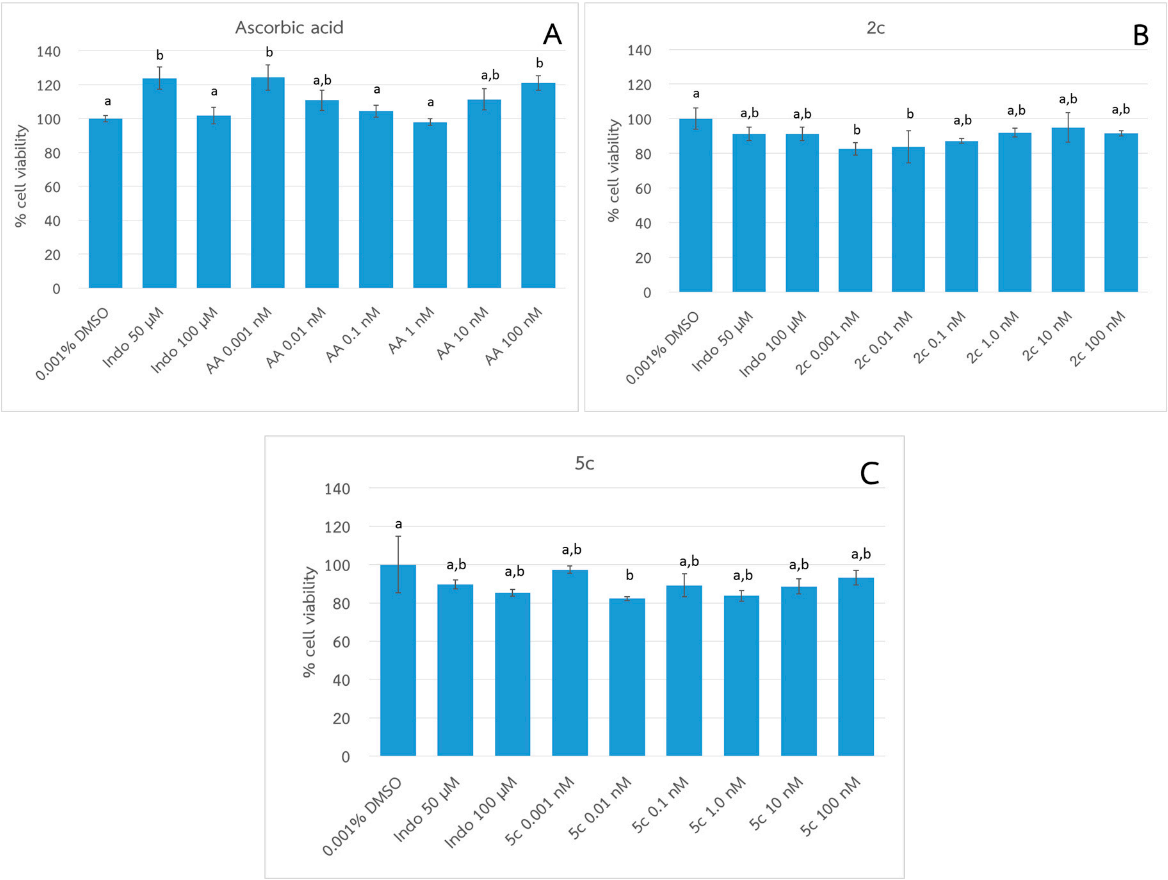

Figure S14. Effects of ascorbic acid (A), **2c** (B) and **5c** (C) on the viability of RAW 264.7. The cytotoxicity was evaluated by MTT assay. Each bar is presented as mean ± SD (n = 3). Letters (a and b) indicate significant differences (p-value < 0.05) based on Tukey's HSD one-way ANOVA by using PAST version 3.14.
